# Neural signature of human–dog interactions: EEG correlates from comparisons with plant and replica dog in a within-subject, cross-over randomized trial

**DOI:** 10.3389/fnhum.2026.1731796

**Published:** 2026-02-06

**Authors:** Fabio Carbone, Caroline Martin-Grieder, Karin Hediger

**Affiliations:** 1 Faculty of Psychology, University of Basel, Basel, Switzerland; 2 REHAB Basel, Basel, Switzerland; 3 Department of Epidemiology and Public Health, Swiss Tropical and Public Health Institute, Allschwil, Switzerland; 4 Faculty of Behavioral Sciences and Psychology, University of Lucerne, Lucerne, Switzerland

**Keywords:** electroencephalography, frequencies analysis, human-animal interaction, neuraloscillation, power spectral density

## Abstract

**Background:**

Human–dog interactions (HDIs) are widely associated with emotional and physiological benefits, yet their neural underpinnings remain poorly understood. This study investigated cortical oscillatory dynamics during HDI compared to two control conditions: interacting with a plant and a realistic dog replica.

**Methods:**

In a within-subject, cross-over randomized design, 29 healthy adults participated in three EEG sessions, each including all conditions. EEG power spectral density was analyzed across theta (4–8 Hz), alpha (8–13 Hz, including slow and fast sub-bands), and beta (13–30 Hz, including low and mid sub-bands) frequencies from 10 electrodes covering prefrontal, frontal, central, parietal, and occipital regions.

**Results:**

Our observations revealed distinct neural signatures for HDI. Compared to plant and replica conditions, dog interaction was characterized by reduced alpha power, particularly in occipital and parietal sites, suggesting increased attentional engagement. Beta power was enhanced in frontal and central electrodes during dog interaction, reflecting heightened cognitive processing and sensorimotor readiness. Theta activity showed regional differences, with lower occipital theta power during HDI relative to controls, indicative of externally oriented attention. By contrast, the replica dog occasionally elicited increased beta and theta power, suggesting compensatory or evaluative processing of ambiguous stimuli.

**Conclusion:**

These findings provide novel electrophysiological evidence that interacting with a real dog elicits unique oscillatory patterns distinct from those observed with non-sentient—living or non-living—controls. The results highlight the social and emotional salience of animals and advance understanding of the neurocognitive mechanisms underlying human–animal interactions, with implications for applied contexts such as animal-assisted treatments.

## 1 Introduction

For many people, interaction with animals brings positive emotions and a sense of wellbeing. As reviewed in Liu et al. (2024), animals have the capacity to reduce negative emotions such as depression, stress, anxiety, or loneliness (Krause-Parello et al., 2019; Janssens et al., 2021; Kogan et al., 2021). In addition, animals can enhance subjective wellbeing, life satisfaction, or other positive states of mind (Bao and Schreer, 2016; Curl et al., 2021; Junça-Silva, 2022). Teo et al. (2022) reviewed the physiological effects of human–dog interaction (HDI), finding mixed results for heart rate and blood pressure, as many studies showed no significant changes. However, most studies reported increased heart rate variability (HRV) and oxytocin levels and decreased cortisol, suggesting stress reduction and improved emotional states. Still, Gandenberger et al. (2022) noted that during HDI, self-reported stress measures often do not align with physiological markers like cortisol. While a good number of psychophysiological parameters have been used to evaluate the impact of human–animal interaction (HAI) on humans, neurological aspects are still largely unexplored. As suggested by several authors, research focusing on neurophysiological processes is needed to better understand the mechanisms underlying HAI (Borgi and Cirulli, 2016; Beetz, 2017; Teo et al., 2022; Liu et al., 2024). Electroencephalography (EEG) is a promising tool for assessing the cognitive and emotional dimensions of HDI.

EEG provides a non-invasive and temporally precise method to investigate neural dynamics associated with mental states during ongoing interaction. Power spectral density (PSD) analysis allows the quantification of oscillatory activity across canonical frequency bands that have been linked to attentional engagement, affective processing, cognitive workload and various emotional states (Chikhi et al., 2022; Xu et al., 2024, 2025; Gkintoni and Halkiopoulos, 2025). These processes are likely to be dynamically modulated during interactive contexts such as HAI, where social presence, touch, and interactional contingency may influence moment-to-moment neural states. EEG is particularly well suited to capture such transient and state-dependent neural changes, as it enables the assessment of continuous brain activity during naturalistic interactions.

To date, only a few studies have investigated the effect of HDI on EEG signals. Calcaterra et al. (2015) used EEG to examine the postoperative benefits of animal-assisted interventions in children. They found that the presence of a dog accelerated the diffusion of beta activity, suggesting facilitated recovery after anesthesia compared to when the dog was not present. Yoo et al. (2024) conducted an EEG study to explore psychophysiological responses to HDI based on what type of activity was undertaken with a dog. They reported increased alpha power (relative slow and fast) during activities such as playing and walking with a dog, potentially reflecting enhanced relaxation. Furthermore, beta power (relative low and mid) was elevated during activities like massaging, grooming, and playing with a dog, which may indicate improved concentration. Another recent study investigated the neurophysiological effect of HDI by measuring EEG power in dog owners. EEG data from a single prefrontal electrode was recorded together with HR and HRV data as well as subjective relaxation during five conditions (baseline resting, relaxation-induction exercise, patting a toy dog, real dog present, and patting a real dog). The authors found higher delta, theta, alpha, and beta power as well as higher HR during the petting activity with a real dog compared to all the other conditions. The results were interpreted as indicating increased relaxation and focused attention during HDI (Teo et al., 2024). A few other studies have investigated how EEG signals were impacted when interacting with animals other than dogs. Cho (2017) found that horseback riding increased relative fast alpha power in elderly participants compared to riding mechanical horses. Homma et al. (2011) reported increased frontal alpha and theta activity in children after dolphin-assisted therapy, though it was unclear if this reflected task-related reductions or posttask enhancements. These changes were negatively correlated with trait anxiety, suggesting a link between increased frontal slow-wave activity and reduced anxiety. Carbone et al. (2025) used a specific EEG feature called frontal alpha asymmetry to measure the objective motivation of healthy participants while they were interacting with a real dog, a replica of a dog, or a plant. Their results showed no differences in objective motivation using this biomarker.

To our knowledge, the effects of HDI on EEG signals have not yet been systematically investigated using an experimental paradigm with carefully matched control conditions. Previous studies have often compared different types of activity, which is problematic because EEG is highly sensitive to the nature of the activity itself. This can lead to misinterpretation of the results. Establishing appropriate control conditions is crucial for isolating the specific neural effects of HDI. To fill this gap, we measured EEG PSD on 10 electrodes during interaction with a real dog compared to an interaction with a replica dog and a plant in 29 healthy participants. PSD measures are relatively robust to short recording segments and compatible with repeated-measures designs, making them appropriate for paradigms involving multiple interaction conditions (Levin et al., 2020). In the present study, EEG PSD was therefore selected to explore potential condition-related modulations of neural activity. Given the exploratory nature of the analysis reported here, we did not establish specific hypotheses.

## 2 Materials and methods

### 2.1 Trial design and procedure

This article reports data from a randomized controlled trial with a within-subject design and repeated measures evaluating the effect of HDI on EEG PSD. The primary outcomes have already been published elsewhere (Carbone et al., 2025).

All the participants followed the same protocol, which consisted of three independent EEG measurement sessions conducted 1 week apart, always scheduled at the same time of day for each individual. During each session, participants were exposed to three distinct conditions: a real dog, a replica dog, and a plant. The order of these conditions was randomized within each session to control for potential sequence effects. Each session began and ended with a baseline EEG recording, during which participants focused on a cross on a wall for 5 min. Then each condition was recorded separately in 5-min EEG blocks. For a more detailed description of the study procedures, see previous publication (Carbone et al., 2025).

### 2.2 Participants

Healthy adult participants were invited to take part in the study. They were recruited through online advertisements and flyers outlining the study design, intervention, and participant responsibilities. Recruitment focused on a gender-balanced sample. The eligibility criteria included being aged 18 or older and capable of providing written informed consent. The exclusion criteria encompassed individuals with a fear of dogs, dog allergies, or acute or chronic medical conditions (e.g., chronic pain, hypertension, heart disease, kidney disease, liver disease, or diabetes). Individuals currently taking medications (e.g., psychoactive drugs, narcotics, analgesics), undergoing psychological or psychiatric treatment, or reporting current or regular drug use (e.g., THC within 24 h before the visit, cocaine, heroin) were also excluded. Written informed consent was obtained from all participants prior to the study start. Recruitment and data collection occurred at the Faculty of Psychology, University of Basel, Switzerland, between April and July 2023. Participants were compensated with 100 Swiss francs for completing the study. Thirty participants were recruited for the study. One participant was excluded from the analysis due to noncompliance with the study protocol. The final sample therefore consisted of 29 participants (mean age = 28.07 years, SD = 10.3; 15 females, 14 males). More details can be found in Carbone et al. (2025).

### 2.3 Intervention

The intervention consisted of the presence of a real dog. *Presence* means that the dog was in the room, and physical contact between the dog and the participant was encouraged whenever possible. This was facilitated by having the dog lie beside the participant while the participant gently petted the dog with their right hand, if the dog did not choose to move away. Participants were seated on a low chair to make contact with the dog easier. It must be noted that the interaction was operationalized as engagement within a shared human–dog context, including co-presence and physical contact whenever possible, in order to preserve the ecological validity of HAI. Due to the nature of the intervention, blinding was not possible as the presence of a dog or one of the control conditions could not be hidden to the participants nor the experimenters.

#### 2.3.1 Dogs

The dogs involved in the study were specifically trained for HDIs and the specific task of this study and were accustomed to working with a variety of individuals. They were also familiarized with the study environment, including the room, materials, and staff. To prioritize their wellbeing and manage their workload, each dog participated in a maximum of three sessions per day, totaling approximately 20 min, and was involved in the intervention no more than 2 days per week. Three dogs took part in the study: a 3-year-old male flat-coated retriever, a 3-year-old female Bernese mountain dog, and a 3-year-old male mixed breed. Each participant was consistently paired with the same dog throughout the study.

#### 2.3.2 Replica dog

As the primary control condition, we used a therapeutic replica dog named *Medor* (Joy for All™, © 2020 Ageless Innovation). This lifelike robotic companion featured realistic fur, the ability to bark, a simulated heartbeat, and responses to touch and voice. Commonly utilized in home care and nursing facilities, the replica dog closely imitated the appearance and interactive behaviors of a real dog. For the interaction, *Medor* was placed next to the participant, and the instructions mirrored those provided for the real-dog condition. Participants were instructed to touch and pet the replica dog using their right hand.

#### 2.3.3 Plant

As an additional control condition, we included a real plant (*Epipremnum aureum*). The plant was chosen to assess engagement with a common nonanimal living object with distinctly different characteristics. This specific plant species has been used in prior research that investigated its effect on electrical activity in the frontal brain (Oh et al., 2019).

Participants were given instructions similar to those for the real-dog condition and were asked to touch the plant with their right hand.

### 2.4 Outcomes

#### 2.4.1 EEG data acquisition and processing

The EEG device used in this study consisted of 64 pin-type active AgCl electrodes manufactured by BioSemi BV, Amsterdam, following the international 10–10 system. To ensure precise placement, the head cap was centered on participants’ heads by aligning Cz with the intersection of the anion-nasion and ear-to-ear axes. Data were recorded using ActiView software (version 9.02, BioSemi BV, Amsterdam) at a sampling rate of 2048 Hz. Electrodes were applied according to the manufacturer’s guidelines, maintaining an electrode offset within ±40 mV. All the conditions, including a baseline and a neutral phase, were recorded within a single session, and recording was briefly paused during transitions to avoid unnecessary data collection. Markers were used to indicate the start and end of each condition.

Postrecording, the data were edited and cropped using ActiRead and ActiTool (version 9.02, BioSemi BV, Amsterdam) to retain only the 5-min exposure segments for each condition. A total of 435 recordings were successfully obtained and formed the basis for further analysis. Data preprocessing was performed in MATLAB (R2022a) using the EEGLAB toolbox (Delorme and Makeig, 2004). Data were imported via the BIOSIG plugin (version 3.8.0) (Schlogl and Brunner, 2008), rereferenced to the average signal, and filtered with basic FIR filters (high pass at 0.5 Hz and low pass at 75 Hz). Major movement artifacts and defective channels were manually removed. Artifact components were identified using independent component analysis (ICA) and removed using the EEGLAB plugin ADJUST (version 1.1.1) (Mognon et al., 2011).

#### 2.4.2 Power spectral density analysis

PSD was calculated for the following frequency bands: theta (4–8 Hz), alpha (8–13 Hz), slow alpha (8–11 Hz), fast alpha (11–13 Hz), beta (13–30 Hz), low beta (13–15 Hz), and mid beta (15–20 Hz). PSD analysis focused on the electrodes Fp1, Fp2, F3, F4, C3, C4, P3, P4, O1, and O2. These electrodes were selected to represent prefrontal, frontal, central, parietal, and occipital scalp regions, corresponding to the classical topographical subdivision commonly used in EEG research. This selection allows for coverage of anterior–posterior cortical activity patterns while limiting the number of comparisons. In addition, this electrode set was chosen to partially replicate the analytical approach used in Yoo et al. (2024). The temporal area was excluded from the analysis because it was contaminated by too many muscle artifacts. The PSD was computed on cleaned datasets using the EEGLAB plugin eegstats (Corcoran et al., 2018; Delorme, 2021).

### 2.5 Sample size

The sample size calculation was based on the primary outcome measure, frontal alpha asymmetry (FAA), used in Carbone et al. (2025), and it was estimated that a sample size of 30 participants was needed.

### 2.6 Randomization

As recruitment progressed, the included participants received an ID number from 1 to 30. The randomization of the 90 sessions was performed before the recruitment of the first participant using an in-house R script. Each three-session set was allocated to the 30 ID numbers.

### 2.7 Statistical methods

PSD for each frequency and electrode of interest was compared across all three experimental conditions. Baseline periods were excluded from the main analysis, as they involved resting states that differed substantially from the active tasks in the experimental conditions. Including them would have introduced task-unrelated changes in brain activity, which were not considered physiologically meaningful for this analysis. For that reason, only the HDI and the two active control conditions were compared. Results from the analysis including baseline data are provided in the online databank mentioned in the data availability statement.

An Linear-mixed-model (LMM) was applied with the package lme4 (Bates et al., 2015) and a pairwise comparison between all the conditions was performed using the emmeans package (version 1.8.7; Lenth and Piaskowski, 2017) with the dog condition as the reference, and Holm correction was applied to control for multiple testing. For the LLMs, *t* values between 1.5 and 2.5 were considered indicators of marginal differences, and *t* values > 2.5 were considered indicators of significant changes. The *p* values were calculated based on a likelihood test and were considered significant when *p* < 0.05. For the pairwise comparisons, the significance level was also set at *p* < 0.05. In the models, PSD was used as response variable and computed separately for each condition and session. Data from all three sessions were entered individually into the linear mixed-effects models, with condition and session included as fixed effects and participant as a random effect. No averaging across sessions was performed prior to statistical analysis. A normality check of the residuals was done by visual inspection of q–q plots and Shapiro test. As effect size, we report estimates together with the standard error and 95% CI and Cohen’s *d* for the pairwise comparisons. The analysis was performed with R Statistical Software (version 4.4.2).

### 2.8 Ethical statement

The study protocol, participant information sheet, informed consent, and all the other documents provided to participants were approved by the local ethics committee, the Ethics Commission Northwest and Central Switzerland (project ID: 2023_00206). The study was also preregistered at clinicaltrials.gov (NCT05837546). All the sessions were conducted in accordance with the guidelines of the International Association for Human–Animal Interaction Organizations (IAHAIO; Jegatheesan et al., 2018) and the principles of the Helsinki Declaration (World Medical Association, 2013).

## 3 Results

### 3.1 Linear-mixed-model results

**Figure 1:**
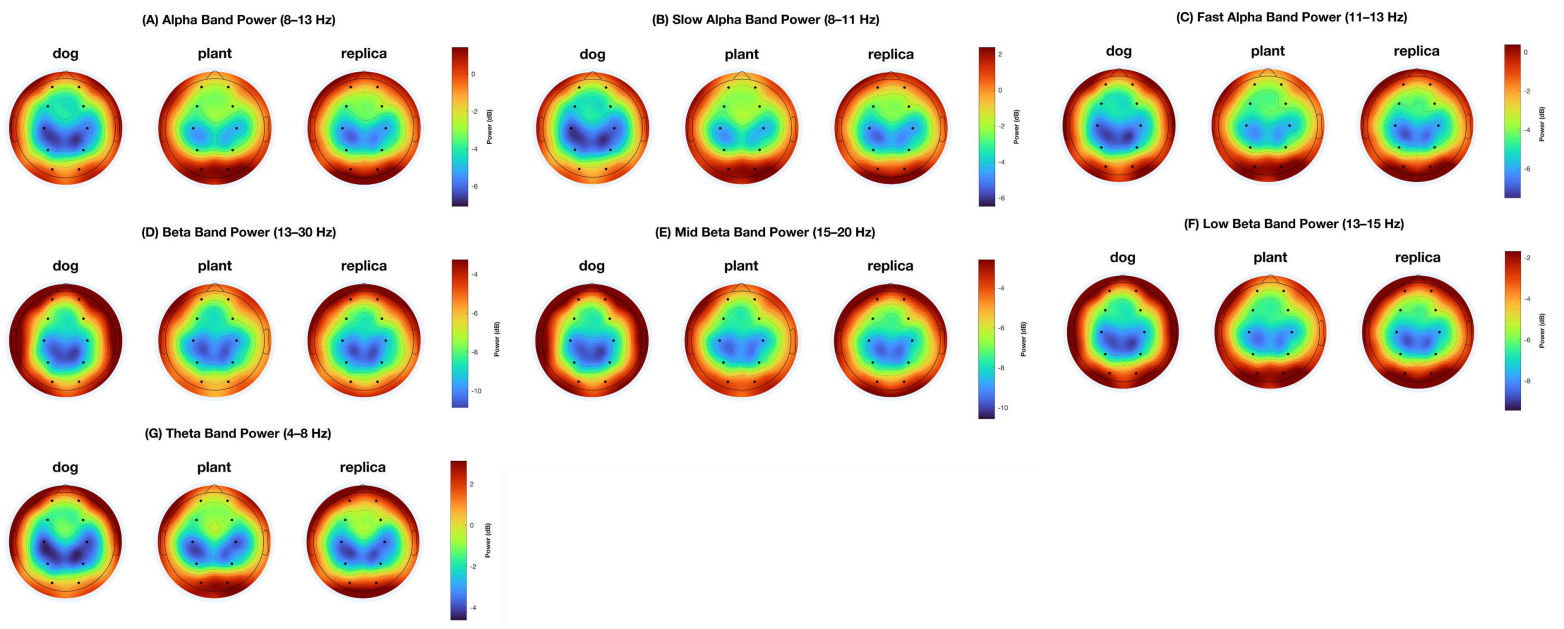
Topographic map of the power spectral density for all frequencies. Each panel displays all three conditions: dog (left), plant (middle), and replica (right). Units are $10\times \log_{10}(\frac{\mu V^{2}}{\mathrm{Hz}})$ . **(A)** alpha (8–13 Hz); **(B)** slow alpha (8–11 Hz); **(C)** fast alpha (11–13 Hz); **(D)** beta (13–30 Hz); **(E)** mid beta (15–20 Hz); **(F)** low beta (13–15 Hz); **(G)** theta (4–8 Hz).

LMM results are shown in Tables 1–7. Figure 1 shows a topographic map of each frequency band per condition. The mean and standard deviation of all the PSDs at each electrode site and for each condition are in Supplementary Table S1.

**Table 1:** Linear-mixed-model results for alpha power at each electrode location.

Channel	Term	Estimate	*SE*	*t* value	*df*	Lower CI	Upper CI	*p* value
Fp1	(Intercept)	−1.28	0.62	−2.07	91	−2.49	−0.07	0.038
	Condition plant	−0.21	0.37	−0.57	229	−0.93	0.51	na
	Condition replica	1.14	0.37	3.12	229	0.43	1.86	na
	Session	−0.32	0.18	−1.73	229	−0.68	0.04	0.083
Fp2	(Intercept)	−1.08	0.66	−1.63	104	−2.38	0.22	0.103
	Condition plant	−0.33	0.41	−0.81	229	−1.14	0.47	na
	Condition replica	0.75	0.41	1.83	229	−0.05	1.56	na
	Session	−0.31	0.21	−1.52	229	−0.72	0.09	0.127
F3	(Intercept)	−3.41	0.53	−6.44	85	−4.44	−2.37	<0.001
	Condition plant	0.33	0.31	1.08	229	−0.27	0.93	na
	Condition replica	0.22	0.31	0.72	229	−0.38	0.82	na
	Session	−0.28	0.15	−1.83	229	−0.58	0.02	0.068
F4	(Intercept)	−3.60	0.56	−6.40	75	−4.71	−2.50	<0.001
	Condition plant	0.56	0.31	1.81	229	−0.05	1.17	na
	Condition replica	0.51	0.31	1.65	229	−0.10	1.12	na
	Session	0.09	0.16	0.57	229	−0.22	0.39	0.567
C3	(Intercept)	−5.78	0.51	−11.25	128	−6.78	−4.77	<0.001
	Condition plant	0.90	0.34	2.66	226	0.24	1.57	na
	Condition replica	0.74	0.34	2.17	226	0.07	1.40	na
	Session	−0.23	0.17	−1.34	226	−0.56	0.10	0.181
C4	(Intercept)	−6.40	0.47	−13.60	91	−7.33	−5.48	<0.001
	Condition plant	0.69	0.28	2.46	229	0.14	1.23	na
	Condition replica	0.40	0.28	1.43	229	−0.15	0.95	na
	Session	0.31	0.14	2.24	229	0.04	0.59	0.025
P3	(Intercept)	−4.92	0.58	−8.53	71	−6.06	−3.79	<0.001
	Condition plant	1.09	0.31	3.49	228	0.48	1.70	na
	Condition replica	0.83	0.31	2.66	228	0.22	1.44	na
	Session	0.00	0.16	0.00	228	−0.30	0.30	1.000
P4	(Intercept)	−5.34	0.64	−8.34	80	−6.59	−4.08	<0.001
	Condition plant	0.82	0.36	2.28	228	0.12	1.53	na
	Condition replica	0.38	0.36	1.05	228	−0.33	1.08	na
	Session	0.42	0.18	2.36	228	0.07	0.78	0.018
O1	(Intercept)	−0.98	0.69	−1.41	62	−2.33	0.38	0.158
	Condition plant	1.45	0.35	4.15	229	0.76	2.13	na
	Condition replica	0.94	0.35	2.70	229	0.26	1.63	na
	Session	0.09	0.17	0.51	229	−0.25	0.43	0.613
O2	(Intercept)	−0.77	0.69	−1.12	63	−2.12	0.58	0.263
	Condition plant	1.24	0.34	3.62	224	0.57	1.92	na
	Condition replica	0.75	0.34	2.19	224	0.08	1.42	na
	Session	0.03	0.17	0.15	224	−0.31	0.36	0.878

Dog is the reference condition. Session effect was also tested. SE = standard error; df = degree of freedom; CI = 95% confidence interval; *p* values < 0.05 were considered significant.

**Table 2:** Linear-mixed-model results for slow alpha power at each electrode location.

Channel	Term	Estimate	*SE*	*t* value	*df*	Lower CI	Upper CI	*p* value
Fp1	(Intercept)	−0.90	0.63	−1.41	92	−2.14	0.35	0.158
	Condition plant	−0.04	0.38	−0.10	229	−0.78	0.70	na
	Condition replica	1.28	0.38	3.39	229	0.54	2.02	na
	Session	−0.30	0.19	−1.57	229	−0.67	0.07	0.115
Fp2	(Intercept)	−0.69	0.67	−1.02	103	−2.01	0.63	0.305
	Condition plant	−0.12	0.42	−0.30	229	−0.94	0.69	na
	Condition replica	0.90	0.42	2.15	229	0.08	1.71	na
	Session	−0.31	0.21	−1.49	229	−0.72	0.10	0.135
F3	(Intercept)	−2.98	0.54	−5.49	85	−4.05	−1.92	<0.001
	Condition plant	0.53	0.31	1.70	229	−0.08	1.15	na
	Condition replica	0.35	0.31	1.12	229	−0.26	0.97	na
	Session	−0.26	0.16	−1.67	229	−0.57	0.05	0.095
F4	(Intercept)	−3.25	0.57	−5.67	75	−4.38	−2.13	<0.001
	Condition plant	0.77	0.32	2.43	229	0.15	1.39	na
	Condition replica	0.66	0.32	2.09	229	0.04	1.28	na
	Session	0.14	0.16	0.87	229	−0.17	0.45	0.385
C3	(Intercept)	−5.53	0.52	−10.74	129	−6.54	−4.52	<0.001
	Condition plant	1.09	0.34	3.20	226	0.42	1.76	na
	Condition replica	0.92	0.34	2.68	226	0.25	1.58	na
	Session	−0.19	0.17	−1.10	226	−0.52	0.15	0.270
C4	(Intercept)	−6.24	0.47	−13.21	93	−7.16	−5.31	<0.001
	Condition plant	0.98	0.28	3.47	229	0.43	1.53	na
	Condition replica	0.66	0.28	2.35	229	0.11	1.22	na
	Session	0.35	0.14	2.48	229	0.07	0.63	0.013
P3	(Intercept)	−4.63	0.59	−7.83	73	−5.79	−3.47	<0.001
	Condition plant	1.31	0.32	4.05	228	0.67	1.94	na
	Condition replica	0.98	0.32	3.02	228	0.34	1.61	na
	Session	0.02	0.16	0.11	228	−0.30	0.33	0.910
P4	(Intercept)	−5.04	0.66	−7.64	78	−6.34	−3.75	<0.001
	Condition plant	1.05	0.37	2.84	228	0.32	1.77	na
	Condition replica	0.55	0.37	1.49	228	−0.17	1.27	na
	Session	0.44	0.18	2.41	228	0.08	0.80	0.016
O1	(Intercept)	−0.78	0.70	−1.12	60	−2.15	0.59	0.264
	Condition plant	1.87	0.34	5.41	229	1.19	2.54	na
	Condition replica	1.19	0.34	3.44	229	0.51	1.86	na
	Session	0.11	0.17	0.63	229	−0.23	0.45	0.530
O2	(Intercept)	−0.51	0.70	−0.73	63	−1.88	0.86	0.466
	Condition plant	1.65	0.35	4.75	224	0.97	2.34	na
	Condition replica	1.02	0.35	2.93	224	0.34	1.70	na
	Session	0.02	0.17	0.11	224	−0.32	0.36	0.910

Dog is the reference condition. Session effect was also tested. SE = standard error; df = degree of freedom; CI = 95% confidence interval; *p* values < 0.05 were considered significant.

**Table 3:** Linear-mixed-model results for fast alpha power at each electrode location.

Channel	Term	Estimate	*SE*	*t* value	*df*	Lower CI	Upper CI	*p* value
Fp1	(Intercept)	−1.93	0.59	−3.27	88	−3.08	−0.77	0.001
	Condition plant	−0.62	0.35	−1.80	229	−1.30	0.06	na
	Condition replica	0.82	0.35	2.37	229	0.14	1.50	na
	Session	−0.37	0.17	−2.15	229	−0.71	−0.03	0.031
Fp2	(Intercept)	−1.72	0.65	−2.65	101	−3.00	−0.45	0.008
	Condition plant	−0.86	0.40	−2.15	229	−1.64	−0.08	na
	Condition replica	0.42	0.40	1.06	229	−0.36	1.21	na
	Session	−0.34	0.20	−1.72	229	−0.74	0.05	0.085
F3	(Intercept)	−4.08	0.52	−7.85	86	−5.10	−3.06	<0.001
	Condition plant	−0.28	0.30	−0.92	229	−0.87	0.31	na
	Condition replica	−0.14	0.30	−0.47	229	−0.74	0.45	na
	Session	−0.34	0.15	−2.27	229	−0.64	−0.05	0.023
F4	(Intercept)	−4.13	0.57	−7.27	74	−5.24	−3.01	<0.001
	Condition plant	−0.08	0.31	−0.25	229	−0.69	0.53	na
	Condition replica	0.11	0.31	0.35	229	−0.50	0.72	na
	Session	−0.05	0.16	−0.30	229	−0.35	0.26	0.766
C3	(Intercept)	−6.15	0.54	−11.35	115	−7.21	−5.09	<0.001
	Condition plant	0.43	0.35	1.24	226	−0.25	1.12	na
	Condition replica	0.37	0.35	1.05	226	−0.32	1.05	na
	Session	−0.30	0.17	−1.72	226	−0.64	0.04	0.085
C4	(Intercept)	−6.58	0.51	−13.00	81	−7.57	−5.59	<0.001
	Condition plant	0.00	0.29	0.01	229	−0.56	0.57	na
	Condition replica	−0.13	0.29	−0.45	229	−0.69	0.43	na
	Session	0.21	0.14	1.47	229	−0.07	0.49	0.141
P3	(Intercept)	−5.42	0.58	−9.42	66	−6.54	−4.29	<0.001
	Condition plant	0.53	0.30	1.76	228	−0.06	1.12	na
	Condition replica	0.47	0.30	1.55	228	−0.12	1.06	na
	Session	−0.03	0.15	−0.22	228	−0.33	0.26	0.830
P4	(Intercept)	−5.85	0.62	−9.38	82	−7.07	−4.63	<0.001
	Condition plant	0.22	0.35	0.62	228	−0.48	0.91	na
	Condition replica	−0.06	0.35	−0.16	228	−0.75	0.64	na
	Session	0.42	0.18	2.39	228	0.08	0.77	0.017
O1	(Intercept)	−1.29	0.68	−1.90	68	−2.63	0.04	0.058
	Condition plant	0.54	0.36	1.51	229	−0.16	1.25	na
	Condition replica	0.46	0.36	1.27	229	−0.25	1.16	na
	Session	0.01	0.18	0.07	229	−0.34	0.36	0.947
O2	(Intercept)	−1.19	0.70	−1.71	61	−2.55	0.17	0.088
	Condition plant	0.34	0.34	1.01	224	−0.32	1.01	na
	Condition replica	0.19	0.34	0.57	224	−0.47	0.86	na
	Session	0.03	0.17	0.15	224	−0.31	0.36	0.880

Dog is the reference condition. Session effect was also tested. SE = standard error; df = degree of freedom; CI = 95% confidence interval; *p* values < 0.05 were considered significant.

**Table 4:** Linear-mixed-model results for beta power at each electrode location.

Channel	Term	Estimate	*SE*	*t* value	*df*	Lower CI	Upper CI	*p* value
Fp1	(Intercept)	−4.04	0.58	−6.98	89	−5.18	−2.91	<0.001
	Condition plant	−1.13	0.34	−3.30	229	−1.79	−0.46	na
	Condition replica	0.33	0.34	0.96	229	−0.34	1.00	na
	Session	−0.48	0.17	−2.82	229	−0.82	−0.15	0.005
Fp2	(Intercept)	−3.81	0.62	−6.10	108	−5.03	−2.59	<0.001
	Condition plant	−1.49	0.39	−3.81	229	−2.26	−0.72	na
	Condition replica	−0.16	0.39	−0.41	229	−0.93	0.61	na
	Session	−0.42	0.20	−2.15	229	−0.80	−0.04	0.032
F3	(Intercept)	−5.93	0.53	−11.24	112	−6.97	−4.90	<0.001
	Condition plant	−0.98	0.33	−2.94	229	−1.64	−0.33	na
	Condition replica	−0.85	0.33	−2.53	229	−1.50	−0.19	na
	Session	−0.49	0.17	−2.90	229	−0.81	−0.16	0.004
F4	(Intercept)	−5.94	0.58	−10.21	92	−7.08	−4.80	<0.001
	Condition plant	−0.95	0.35	−2.73	229	−1.63	−0.27	na
	Condition replica	−0.70	0.35	−2.02	229	−1.38	−0.02	na
	Session	−0.23	0.17	−1.32	229	−0.57	0.11	0.187
C3	(Intercept)	−8.24	0.53	−15.52	126	−9.28	−7.20	<0.001
	Condition plant	−0.65	0.35	−1.87	226	−1.34	0.03	na
	Condition replica	−0.27	0.35	−0.78	226	−0.96	0.41	na
	Session	−0.43	0.17	−2.50	226	−0.77	−0.09	0.013
C4	(Intercept)	−8.71	0.52	−16.64	118	−9.74	−7.69	<0.001
	Condition plant	−1.09	0.34	−3.24	229	−1.75	−0.43	na
	Condition replica	−0.89	0.34	−2.63	229	−1.55	−0.23	na
	Session	0.06	0.17	0.36	229	−0.27	0.39	0.715
P3	(Intercept)	−8.38	0.45	−18.77	101	−9.26	−7.51	<0.001
	Condition plant	−0.34	0.28	−1.24	228	−0.88	0.20	na
	Condition replica	−0.20	0.28	−0.73	228	−0.74	0.34	na
	Session	−0.19	0.14	−1.38	228	−0.46	0.08	0.167
P4	(Intercept)	−8.80	0.54	−16.22	116	−9.86	−7.74	<0.001
	Condition plant	−0.58	0.35	−1.68	228	−1.26	0.10	na
	Condition replica	−0.63	0.35	−1.82	228	−1.30	0.05	na
	Session	0.15	0.17	0.85	228	−0.19	0.48	0.397
O1	(Intercept)	−4.90	0.51	−9.67	84	−5.89	−3.90	<0.001
	Condition plant	−0.38	0.29	−1.29	229	−0.95	0.20	na
	Condition replica	−0.40	0.29	−1.38	229	−0.98	0.17	na
	Session	−0.20	0.15	−1.35	229	−0.48	0.09	0.177
O2	(Intercept)	−5.17	0.54	−9.50	61	−6.24	−4.11	<0.001
	Condition plant	−0.52	0.27	−1.93	224	−1.04	0.01	na
	Condition replica	−0.42	0.27	−1.57	224	−0.95	0.10	na
	Session	−0.16	0.13	−1.19	224	−0.42	0.10	0.232

Dog is the reference condition. Session effect was also tested. SE = standard error; df = degree of freedom; CI = 95% confidence interval; *p* values < 0.05 were considered significant.

**Table 5:** Linear-mixed-model results for low beta power at each electrode location.

Channel	Term	Estimate	*SE*	*t* value	*df*	Lower CI	Upper CI	*p* value
Fp1	(Intercept)	−2.65	0.57	−4.62	87	−3.78	−1.53	<0.001
	Condition plant	−0.79	0.34	−2.36	229	−1.45	−0.13	na
	Condition replica	0.74	0.34	2.20	229	0.08	1.40	na
	Session	−0.47	0.17	−2.79	229	−0.80	−0.14	0.005
Fp2	(Intercept)	−2.42	0.64	−3.80	103	−3.66	−1.17	<0.001
	Condition plant	−1.10	0.39	−2.79	229	−1.87	−0.33	na
	Condition replica	0.28	0.39	0.72	229	−0.49	1.05	na
	Session	−0.43	0.20	−2.20	229	−0.82	−0.05	0.028
F3	(Intercept)	−4.71	0.48	−9.76	94	−5.66	−3.76	<0.001
	Condition plant	−0.56	0.29	−1.92	229	−1.13	0.01	na
	Condition replica	−0.36	0.29	−1.24	229	−0.93	0.21	na
	Session	−0.47	0.15	−3.26	229	−0.76	−0.19	0.001
F4	(Intercept)	−4.82	0.55	−8.79	81	−5.90	−3.75	<0.001
	Condition plant	−0.40	0.31	−1.29	229	−1.01	0.21	na
	Condition replica	−0.06	0.31	−0.19	229	−0.67	0.55	na
	Session	−0.17	0.16	−1.08	229	−0.47	0.14	0.282
C3	(Intercept)	−6.93	0.50	−13.78	122	−7.91	−5.94	<0.001
	Condition plant	0.10	0.33	0.30	226	−0.54	0.74	na
	Condition replica	0.16	0.33	0.49	226	−0.48	0.80	na
	Session	−0.39	0.16	−2.38	226	−0.71	−0.07	0.017
C4	(Intercept)	−7.51	0.46	−16.26	89	−8.42	−6.61	<0.001
	Condition plant	−0.23	0.27	−0.83	229	−0.76	0.31	na
	Condition replica	−0.22	0.27	−0.80	229	−0.75	0.32	na
	Session	0.13	0.14	0.92	229	−0.14	0.39	0.357
P3	(Intercept)	−6.28	0.49	−12.81	73	−7.25	−5.32	<0.001
	Condition plant	0.24	0.27	0.89	228	−0.29	0.76	na
	Condition replica	0.29	0.27	1.06	228	−0.24	0.81	na
	Session	−0.16	0.13	−1.19	228	−0.42	0.10	0.234
P4	(Intercept)	−6.80	0.56	−12.12	98	−7.90	−5.70	<0.001
	Condition plant	0.02	0.34	0.05	228	−0.65	0.68	na
	Condition replica	−0.15	0.34	−0.45	228	−0.82	0.51	na
	Session	0.28	0.17	1.65	228	−0.05	0.61	0.100
O1	(Intercept)	−2.27	0.59	−3.85	72	−3.43	−1.12	<0.001
	Condition plant	0.28	0.32	0.88	229	−0.34	0.91	na
	Condition replica	0.27	0.32	0.83	229	−0.36	0.89	na
	Session	−0.10	0.16	−0.65	229	−0.42	0.21	0.516
O2	(Intercept)	−2.32	0.61	−3.81	58	−3.51	−1.12	<0.001
	Condition plant	0.17	0.29	0.60	224	−0.39	0.74	na
	Condition replica	0.15	0.29	0.50	224	−0.42	0.71	na
	Session	−0.09	0.15	−0.62	224	−0.37	0.19	0.536

Dog is the reference condition. Session effect was also tested. SE = standard error; df = degree of freedom; CI = 95% confidence interval; *p* values < 0.05 were considered as significant.

**Table 6:** Linear-mixed-model results for mid beta power at each electrode location.

Channel	Term	Estimate	*SE*	*t* value	*df*	Lower CI	Upper CI	*p* value
Fp1	(Intercept)	−3.62	0.57	−6.30	87	−4.75	−2.50	<0.001
	Condition plant	−0.97	0.34	−2.89	229	−1.63	−0.31	na
	Condition replica	0.51	0.34	1.53	229	−0.14	1.17	na
	Session	−0.48	0.17	−2.88	229	−0.81	−0.15	0.004
Fp2	(Intercept)	−3.39	0.62	−5.44	104	−4.61	−2.17	<0.001
	Condition plant	−1.32	0.39	−3.42	229	−2.08	−0.56	na
	Condition replica	−0.03	0.39	−0.08	229	−0.79	0.73	na
	Session	−0.42	0.19	−2.16	229	−0.80	−0.04	0.031
F3	(Intercept)	−5.49	0.51	−10.79	108	−6.49	−4.49	<0.001
	Condition plant	−0.80	0.32	−2.50	229	−1.43	−0.17	na
	Condition replica	−0.67	0.32	−2.09	229	−1.29	−0.04	na
	Session	−0.49	0.16	−3.04	229	−0.80	−0.17	0.002
F4	(Intercept)	−5.59	0.57	−9.81	86	−6.71	−4.47	<0.001
	Condition plant	−0.69	0.33	−2.08	229	−1.34	−0.04	na
	Condition replica	−0.44	0.33	−1.33	229	−1.09	0.21	na
	Session	−0.20	0.17	−1.19	229	−0.52	0.13	0.235
C3	(Intercept)	−7.95	0.51	−15.44	129	−8.95	−6.94	<0.001
	Condition plant	−0.38	0.34	−1.13	226	−1.05	0.28	na
	Condition replica	0.00	0.34	−0.01	226	−0.67	0.67	na
	Session	−0.37	0.17	−2.20	226	−0.71	−0.04	0.028
C4	(Intercept)	−8.52	0.48	−17.72	122	−9.46	−7.58	<0.001
	Condition plant	−0.63	0.31	−2.02	229	−1.24	−0.02	na
	Condition replica	−0.43	0.31	−1.38	229	−1.04	0.18	na
	Session	0.12	0.16	0.79	229	−0.18	0.43	0.430
P3	(Intercept)	−7.79	0.45	−17.45	99	−8.66	−6.91	<0.001
	Condition plant	0.00	0.27	−0.01	228	−0.54	0.53	na
	Condition replica	0.11	0.27	0.39	228	−0.43	0.64	na
	Session	−0.17	0.14	−1.21	228	−0.43	0.10	0.226
P4	(Intercept)	−8.20	0.54	−15.21	114	−9.25	−7.14	<0.001
	Condition plant	−0.25	0.34	−0.75	228	−0.92	0.41	na
	Condition replica	−0.32	0.34	−0.94	228	−0.99	0.35	na
	Session	0.17	0.17	0.98	228	−0.17	0.50	0.327
O1	(Intercept)	−4.27	0.50	−8.50	81	−5.25	−3.28	<0.001
	Condition plant	0.06	0.29	0.20	229	−0.50	0.62	na
	Condition replica	−0.03	0.29	−0.12	229	−0.59	0.53	na
	Session	−0.19	0.14	−1.29	229	−0.47	0.10	0.195
O2	(Intercept)	−4.45	0.55	−8.14	58	−5.52	−3.38	<0.001
	Condition plant	−0.08	0.26	−0.30	224	−0.59	0.43	na
	Condition replica	−0.07	0.26	−0.28	224	−0.59	0.44	na
	Session	−0.18	0.13	−1.36	224	−0.44	0.08	0.173

Dog is the reference condition. Session effect was also tested. SE = standard error; df = degree of freedom; CI = 95% confidence interval; *p* values < 0.05 were considered significant.

**Table 7:** Linear-mixed-model results for theta power at each electrode location.

Channel	Term	Estimate	*SE*	*t* value	*df*	Lower CI	Upper CI	*p* value
Fp1	(Intercept)	2.10	0.69	3.03	92	0.74	3.45	0.002
	Condition plant	−0.84	0.41	−2.04	229	−1.65	−0.03	na
	Condition replica	1.09	0.41	2.64	229	0.28	1.90	na
	Session	−0.66	0.21	−3.21	229	−1.07	−0.26	0.001
Fp2	(Intercept)	2.16	0.72	3.02	97	0.76	3.56	0.003
	Condition plant	−0.94	0.43	−2.16	229	−1.79	−0.09	na
	Condition replica	0.68	0.43	1.56	229	−0.17	1.53	na
	Session	−0.62	0.22	−2.86	229	−1.04	−0.20	0.004
F3	(Intercept)	−0.27	0.49	−0.55	102	−1.22	0.68	0.580
	Condition plant	−0.37	0.30	−1.23	229	−0.96	0.22	na
	Condition replica	0.06	0.30	0.20	229	−0.53	0.65	na
	Session	−0.58	0.15	−3.87	229	−0.88	−0.29	<0.001
F4	(Intercept)	−0.84	0.52	−1.63	105	−1.85	0.17	0.102
	Condition plant	−0.13	0.32	−0.40	229	−0.76	0.50	na
	Condition replica	0.46	0.32	1.43	229	−0.17	1.09	na
	Session	−0.09	0.16	−0.57	229	−0.41	0.22	0.571
C3	(Intercept)	−3.33	0.50	−6.61	155	−4.32	−2.34	<0.001
	Condition plant	0.27	0.35	0.77	226	−0.42	0.96	na
	Condition replica	0.66	0.35	1.90	226	−0.02	1.35	na
	Session	−0.39	0.17	−2.23	226	−0.73	−0.05	0.026
C4	(Intercept)	−4.31	0.42	−10.22	141	−5.13	−3.48	<0.001
	Condition plant	0.14	0.28	0.50	229	−0.41	0.70	na
	Condition replica	0.43	0.28	1.52	229	−0.13	0.99	na
	Session	0.15	0.14	1.04	229	−0.13	0.43	0.297
P3	(Intercept)	−2.76	0.45	−6.17	143	−3.63	−1.88	<0.001
	Condition plant	0.39	0.30	1.27	228	−0.21	0.98	na
	Condition replica	0.49	0.30	1.61	228	−0.11	1.09	na
	Session	−0.25	0.15	−1.63	228	−0.55	0.05	0.103
P4	(Intercept)	−3.33	0.57	−5.85	157	−4.45	−2.22	<0.001
	Condition plant	0.12	0.39	0.30	228	−0.65	0.89	na
	Condition replica	0.01	0.39	0.03	228	−0.76	0.78	na
	Session	0.26	0.20	1.33	228	−0.12	0.65	0.184
O1	(Intercept)	0.88	0.50	1.76	86	−0.10	1.87	0.079
	Condition plant	1.22	0.29	4.16	229	0.64	1.79	na
	Condition replica	1.03	0.29	3.51	229	0.45	1.60	na
	Session	−0.19	0.15	−1.28	229	−0.47	0.10	0.200
O2	(Intercept)	0.96	0.52	1.86	67	−0.05	1.98	0.063
	Condition plant	1.31	0.27	4.90	224	0.78	1.83	na
	Condition replica	1.08	0.27	4.03	224	0.55	1.60	na
	Session	−0.24	0.13	−1.75	224	−0.50	0.03	0.079

Dog is the reference condition. Session effect was also tested. SE = standard error; df = degree of freedom; CI = 95% confidence interval; *p* values < 0.05 were considered as significant.

#### 3.1.1 Alpha

Alpha power analyses revealed condition-dependent effects that varied by scalp region. At the frontal electrodes (Fp1, Fp2, F3, F4), no significant differences emerged between the conditions (all *p* > 0.05), except at Fp1, where alpha power was higher in the replica condition compared to the dog condition (*b* = 1.14, *SE* = 0.37, *t*(229) = 3.12, 95% CI [0.43, 1.86]). In contrast, robust effects were observed at more posterior sites. At the central electrodes, alpha power was greater in the plant condition compared to the dog at C3 (*b* = 0.90, *SE* = 0.34, *t*(226) = 2.66, 95% CI [0.24, 1.57]) and at C4 (*b* = 0.69, *SE* = 0.28, *t*(229) = 2.46, 95% CI [0.14, 1.23]). Similar effects were present at the parietal electrodes: at P3, both the plant (*b* = 1.09, *SE* = 0.31, *t*(228) = 3.49, 95% CI [0.48, 1.70]) and the replica conditions (*b* = 0.83, *SE* = 0.31, *t*(228) = 2.66, 95% CI [0.22, 1.44]) showed higher alpha power relative to the dog. At P4, only the plant condition was significantly higher than the dog (*b* = 0.82, *SE* = 0.36, *t*(228) = 2.28, 95% CI [0.12, 1.53]). The strongest effects were observed at the occipital electrodes: at O1, both the plant (*b* = 1.45, *SE* = 0.35, *t*(229) = 4.15, 95% CI [0.76, 2.13]) and the replica (*b* = 0.94, *SE* = 0.35, *t*(229) = 2.70, 95% CI [0.26, 1.63]) conditions elicited greater alpha power than the dog, with a similar pattern at O2 (plant: *b* = 1.24, *SE* = 0.34, *t*(224) = 3.62, 95% CI [0.57, 1.92]; replica: *b* = 0.75, *SE* = 0.34, *t*(224) = 2.19, 95% CI [0.08, 1.42]). Session effects were modest and inconsistent, reaching significance at C4 (*b* = 0.31, *SE* = 0.14, *t*(229) = 2.24, 95% CI [0.04, 0.59]) and P4 (*b* = 0.42, *SE* = 0.18, *t*(228) = 2.36, 95% CI [0.07, 0.78]).

#### 3.1.2 Slow alpha

Analyses of slow alpha power revealed condition effects that were most pronounced in the central, parietal, and occipital regions. At the frontal electrodes, no consistent differences were observed between the conditions, although alpha was higher in the replica compared to the dog condition at Fp1 (*b* = 1.28, *SE* = 0.38, *t*(229) = 3.39, 95% CI [0.54, 2.02]) and Fp2 (*b* = 0.90, *SE* = 0.42, *t*(229) = 2.15, 95% CI [0.08, 1.71]). More robust effects emerged over the central electrodes: both the plant (*b* = 1.09, *SE* = 0.34, *t*(226) = 3.20, 95% CI [0.42, 1.76]) and the replica (*b* = 0.92, *SE* = 0.34, *t*(226) = 2.68, 95% CI [0.25, 1.58]) conditions showed greater alpha relative to the dog at C3, with a similar pattern at C4 (plant: *b* = 0.98, *SE* = 0.28, *t*(229) = 3.47, 95% CI [0.43, 1.53]; replica: *b* = 0.66, *SE* = 0.28, *t*(229) = 2.35, 95% CI [0.11, 1.22]). At the parietal electrodes, alpha power was significantly higher in the plant condition compared to the dog at both P3 (*b* = 1.31, *SE* = 0.32, *t*(228) = 4.05, 95% CI [0.67, 1.94]) and P4 (*b* = 1.05, *SE* = 0.37, *t*(228) = 2.84, 95% CI [0.32, 1.77]), while the replica condition was significant at P3 (*b* = 0.98, *SE* = 0.32, *t*(228) = 3.02, 95% CI [0.34, 1.61]) but not P4. The strongest condition differences were observed at the occipital electrodes: the plant (*b* = 1.87, *SE* = 0.34, *t*(229) = 5.41, 95% CI [1.19, 2.54]) and replica conditions (*b* = 1.19, *SE* = 0.34, *t*(229) = 3.44, 95% CI [0.51, 1.86]) both showed greater alpha than the dog at O1, with a similar pattern at O2 (plant: *b* = 1.65, *SE* = 0.35, *t*(224) = 4.75, 95% CI [0.97, 2.34]; replica: *b* = 1.02, *SE* = 0.35, *t*(224) = 2.93, 95% CI [0.34, 1.70]). Session effects were modest, reaching significance at C4 (*b* = 0.35, *SE* = 0.14, *t*(229) = 2.48, 95% CI [0.07, 0.63]) and P4 (*b* = 0.44, *SE* = 0.18, *t*(228) = 2.41, 95% CI [0.08, 0.80]), but were otherwise inconsistent.

#### 3.1.3 Fast alpha

Analyses of fast alpha power revealed more limited condition effects compared to total and slow alpha. At the frontal sites, the replica condition elicited greater fast alpha power than the dog condition at Fp1 (*b* = 0.82, *SE* = 0.35, *t*(229) = 2.37, 95% CI [0.14, 1.50]), whereas the plant condition elicited lower fast alpha at Fp2 (*b* = −0.86, *SE* = 0.40, *t*(229) = −2.15, 95% CI [−1.64, −0.08]). No other frontal effects reached significance. At the central, parietal, and occipital electrodes, no reliable differences between conditions were observed. Session effects indicated modest decreases in alpha across repetitions at Fp1 (*b* = −0.37, *SE* = 0.17, *t*(229) = −2.15, 95% CI [−0.71, −0.03]) and F3 (*b* = −0.34, *SE* = 0.15, *t*(229) = −2.27, 95% CI [−0.64, −0.05]), alongside a modest increase at P4 (*b* = 0.42, *SE* = 0.18, *t*(228) = 2.39, 95% CI [0.08, 0.77]).

#### 3.1.4 Beta

For beta activity, widespread condition effects were observed across the frontal and central electrodes. At the frontal sites, both Fp1 and Fp2 showed significantly reduced beta power in the plant condition compared to the dog condition (Fp1: *b* = −1.13, *SE* = 0.34, *t*(229) = −3.30, 95% CI [−1.79, −0.46]; Fp2: *b* = −1.49, *SE* = 0.39, *t*(229) = −3.81, 95% CI [−2.26, −0.72]). Similarly, electrodes F3 (*b* = −0.98, *SE* = 0.33, *t*(229) = −2.94, 95% CI [−1.64, −0.33]) and F4 (*b* = −0.95, *SE* = 0.35, *t*(229) = −2.73, 95% CI [−1.63, −0.27]) showed lower beta power in the plant condition, with additional decreases in the replica condition (F3: *b* = −0.85, *SE* = 0.33, *t*(229) = −2.53, 95% CI [−1.50, −0.19]; F4: *b* = −0.70, *SE* = 0.35, *t*(229) = −2.02, 95% CI [−1.38, −0.02]). The central electrodes displayed similar effects, C4 showing reduced beta power in both the plant (*b* = −1.09, *SE* = 0.34, *t*(229) = −3.24, 95% CI [−1.75, −0.43]) and the replica (*b* = −0.89, *SE* = 0.34, *t*(229) = −2.63, 95% CI [−1.55, −0.23]) conditions, while C3 showed a weaker, nonsignificant decrease in the plant condition (*b* = −0.65, *SE* = 0.35, *t*(226) = −1.87, 95% CI [−1.34, 0.03]). The parietal and occipital electrodes revealed smaller and mostly nonsignificant trends, though O2 showed a marginal reduction in the plant condition (*b* = −0.52, *SE* = 0.27, *t*(224) = −1.93, 95% CI [−1.04, 0.01]). Session effects were also present, as beta power decreased across sessions at several frontal and central electrodes (Fp1: *b* = −0.48, *SE* = 0.17, *t*(229) = −2.82, 95% CI [−0.82, −0.15]; Fp2: *b* = −0.42, *SE* = 0.20, *t*(229) = −2.15, 95% CI [−0.80, −0.04]; F3: *b* = −0.49, *SE* = 0.17, *t*(229) = −2.90, 95% CI [−0.81, −0.16]; C3: *b* = −0.43, *SE* = 0.17, *t*(226) = −2.50, 95% CI [−0.77, −0.09]).

#### 3.1.5 Low beta

For low beta activity, significant effects of condition were most pronounced at the frontal electrodes. At Fp1, beta power was reduced in the plant condition compared to the dog condition (*b* = −0.79, *SE* = 0.34, *t*(229) = −2.36, 95% CI [−1.45, −0.13]) and increased in the replica condition (*b* = 0.74, *SE* = 0.34, *t*(229) = 2.20, 95% CI [0.08, 1.40]). A similar reduction in the plant condition was found at Fp2 (*b* = −1.10, *SE* = 0.39, *t*(229) = −2.79, 95% CI [−1.87, −0.33]). At F3, there was a marginal decrease in the plant condition (*b* = −0.56, *SE* = 0.29, *t*(229) = −1.92, 95% CI [−1.13, 0.01]). In the central regions, C3 showed no condition differences, whereas C4 revealed no significant effects. The posterior electrodes (P3, P4, O1, O2) did not show condition-related modulations. Session effects were also observed, as low beta power decreased over time at Fp1 (*b* = −0.47, *SE* = 0.17, *t*(229) = −2.79, 95% CI [−0.80, −0.14]), Fp2 (*b* = −0.43, *SE* = 0.20, *t*(229) = −2.20, 95% CI [−0.82, −0.05]), F3 (*b* = −0.47, *SE* = 0.15, *t*(229) = −3.26, 95% CI [−0.76, −0.19]), and C3 (*b* = −0.39, *SE* = 0.16, *t*(226) = −2.38, 95% CI [−0.71, −0.07]).

#### 3.1.6 Mid beta

Analyses of mid beta power revealed condition effects that were most pronounced in the frontal and central regions. At the frontal electrodes, activity was lower in the plant condition compared to the dog at Fp1 (*b* = −0.97, *SE* = 0.34, *t*(229) = −2.89, 95% CI [−1.63, −0.31]) and Fp2 (*b* = −1.32, *SE* = 0.39, *t*(229) = −3.42, 95% CI [−2.08, −0.56]), while the replica condition showed smaller, nonsignificant differences (Fp1: *b* = 0.51, *SE* = 0.34, *t*(229) = 1.53, 95% CI [−0.14, 1.17]; Fp2: *b* = −0.03, *SE* = 0.39, *t*(229) = −0.08, 95% CI [−0.79, 0.73]). More pronounced condition differences emerged in the central electrodes: both the plant (*b* = −0.80, *SE* = 0.32, *t*(229) = −2.50, 95% CI [−1.43, −0.17]) and the replica (*b* = −0.67, *SE* = 0.32, *t*(229) = −2.09, 95% CI [−1.29, −0.04]) conditions were associated with lower mid beta at F3, with a similar pattern at F4 for the plant condition (*b* = −0.69, *SE* = 0.33, *t*(229) = −2.08, 95% CI [−1.34, −0.04]) but not the replica condition. Session-related decreases were observed at C3 (*b* = −0.37, *SE* = 0.17, *t*(226) = −2.20, 95% CI [−0.71, −0.04]), and the plant condition showed reduced activity at C4 (*b* = −0.63, *SE* = 0.31, *t*(229) = −2.02, 95% CI [−1.24, −0.02]). Condition and session effects were less pronounced at the parietal (P3, P4) and occipital (O1, O2) electrodes.

#### 3.1.7 Theta

Analyses of theta power revealed condition effects that were most pronounced at the frontal and occipital electrodes. At the frontal electrodes, theta was lower in the plant condition compared to the dog at Fp1 (*b* = −0.84, *SE* = 0.41, *t*(229) = −2.04, 95% CI [−1.65, −0.03]) and Fp2 (*b* = −0.94, *SE* = 0.43, *t*(229) = −2.16, 95% CI [−1.79, −0.09]), while the replica condition showed higher theta at Fp1 (*b* = 1.09, *SE* = 0.41, *t*(229) = 2.64, 95% CI [0.28, 1.90]) and a nonsignificant increase at Fp2 (*b* = 0.68, *SE* = 0.43, *t*(229) = 1.56, 95% CI [−0.17, 1.53]). The central electrodes showed moderate increases in theta for the replica condition at C3 (*b* = 0.66, *SE* = 0.35, *t*(226) = 1.90, 95% CI [−0.02, 1.35]) and a session-related decrease at C3 (*b* = −0.39, *SE* = 0.17, *t*(226) = −2.23, 95% CI [−0.73, −0.05]). At the occipital electrodes, both the plant (O1: *b* = 1.22, *SE* = 0.29, *t*(229) = 4.16, 95% CI [0.64, 1.79]; O2: *b* = 1.31, *SE* = 0.27, *t*(224) = 4.90, 95% CI [0.78, 1.83]) and the replica (O1: *b* = 1.03, *SE* = 0.29, *t*(229) = 3.51, 95% CI [0.45, 1.60]; O2: *b* = 1.08, *SE* = 0.27, *t*(224) = 4.03, 95% CI [0.55, 1.60]) conditions showed higher theta relative to the dog condition; session effects were small and nonsignificant at these sites. Session effects were also present at Fp1 (*b* = −0.66, *SE* = 0.21, *t*(229) = −3.21, 95% CI [−1.07, −0.26]) and Fp2 (*b* = −0.62, *SE* = 0.22, *t*(229) = −2.86, 95% CI [−1.04, −0.20]).

Visual inspection of the scalp topographies (Figure 1) shows spatial patterns that are consistent with the statistical results. In particular, the dog condition is characterized by increased beta activity over frontal regions and reduced alpha power over occipital and central electrodes relative to control conditions. These topographical maps are provided for illustrative purposes only and do not constitute an independent statistical test, but they support the spatial distribution of effects identified in the quantitative analyses.

### 3.2 Pairwise-analysis results

Significant pairwise comparisons are described and presented in Table 8 and illustrated in Supplementary Figure S1.

**Table 8:** Significant pairwise comparisons between the conditions real dog, plant, and replica dog for each frequency and electrode.

Channel	Contrast	*b*	*SE*(*x*)	*df*(*x*)	*t* value	*p* value	ES	*SE*(*y*)	*df*(*y*)	Lower CI	Upper CI
Alpha											
Fp1	dog–replica	−1.14	0.37	229	−3.12	0.004	−0.47	0.15	41	−0.78	−0.16
	plant–replica	−1.35	0.37	229	−3.70	0.001	−0.56	0.15	41	−0.87	−0.25
Fp2	plant–replica	−1.08	0.41	229	−2.64	0.026	−0.40	0.15	44	−0.71	−0.09
C3	dog–plant	−0.90	0.34	226	−2.66	0.025	−0.41	0.15	50	−0.71	−0.10
C4	dog–plant	−0.69	0.28	229	−2.46	0.043	−0.37	0.15	41	−0.68	−0.07
P3	dog–plant	−1.09	0.31	228	−3.49	0.002	−0.53	0.15	37	−0.84	−0.22
	dog–replica	−0.83	0.31	228	−2.66	0.017	−0.40	0.15	37	−0.71	−0.09
O1	dog–plant	−1.45	0.35	229	−4.15	<0.001	−0.63	0.15	36	−0.94	−0.32
	dog–replica	−0.94	0.35	229	−2.70	0.015	−0.41	0.15	36	−0.72	−0.10
O2	dog–plant	−1.24	0.34	224	−3.62	0.001	−0.56	0.15	35	−0.87	−0.24
Slow alpha											
Fp1	dog–replica	−1.28	0.38	229	−3.39	0.002	−0.51	0.15	42	−0.82	−0.20
	plant–replica	−1.32	0.38	229	−3.49	0.002	−0.53	0.15	42	−0.84	−0.22
Fp2	plant–replica	−1.02	0.42	229	−2.45	0.045	−0.37	0.15	44	−0.68	−0.06
F4	dog–plant	−0.77	0.32	229	−2.43	0.047	−0.37	0.15	38	−0.68	−0.06
C3	dog–plant	−1.09	0.34	226	−3.20	0.005	−0.49	0.15	50	−0.80	−0.18
	dog–replica	−0.92	0.34	226	−2.68	0.016	−0.41	0.15	50	−0.72	−0.10
C4	dog–plant	−0.98	0.28	229	−3.47	0.002	−0.53	0.15	42	−0.84	−0.22
	dog–replica	−0.66	0.28	229	−2.35	0.039	−0.36	0.15	42	−0.66	−0.05
P3	dog–plant	−1.31	0.32	228	−4.05	<0.001	−0.61	0.15	38	−0.93	−0.30
	dog–replica	−0.98	0.32	228	−3.02	0.006	−0.46	0.15	38	−0.77	−0.15
P4	dog–plant	−1.05	0.37	228	−2.84	0.015	−0.43	0.15	39	−0.74	−0.12
O1	dog–plant	−1.87	0.34	229	−5.41	<0.001	−0.82	0.15	35	−1.13	−0.51
	dog–replica	−1.19	0.34	229	−3.44	0.001	−0.52	0.15	35	−0.83	−0.21
	plant–replica	0.68	0.34	229	1.97	0.050	0.30	0.15	35	−0.01	0.61
O2	dog–plant	−1.65	0.35	224	−4.75	<0.001	−0.73	0.15	35	−1.04	−0.42
	dog–replica	−1.02	0.35	224	−2.93	0.007	−0.45	0.15	35	−0.76	−0.14
Fast alpha											
Fp1	dog–replica	−0.82	0.35	229	−2.37	0.037	−0.36	0.15	41	−0.67	−0.05
	plant–replica	−1.44	0.35	229	−4.17	<0.001	−0.63	0.15	41	−0.94	−0.32
Fp2	plant–replica	−1.28	0.40	229	−3.21	0.005	−0.49	0.15	44	−0.79	−0.18
Beta											
Fp1	dog–plant	1.13	0.34	229	3.30	0.002	0.50	0.15	41	0.19	0.81
	plant–replica	−1.45	0.34	229	−4.26	<0.001	−0.65	0.15	41	−0.96	−0.33
Fp2	dog–plant	1.49	0.39	229	3.81	0.001	0.58	0.15	45	0.27	0.89
	plant–replica	−1.33	0.39	229	−3.40	0.002	−0.52	0.15	45	−0.82	−0.21
F3	dog–plant	0.98	0.33	229	2.94	0.011	0.45	0.15	46	0.14	0.75
	dog–replica	0.85	0.33	229	2.53	0.024	0.38	0.15	46	0.08	0.69
F4	dog–plant	0.95	0.35	229	2.73	0.020	0.41	0.15	42	0.11	0.72
C4	dog–plant	1.09	0.34	229	3.24	0.004	0.49	0.15	47	0.18	0.80
	dog–replica	0.89	0.34	229	2.63	0.018	0.40	0.15	47	0.09	0.71
Low beta											
Fp1	dog–plant	0.79	0.34	229	2.36	0.038	0.36	0.15	41	0.05	0.67
	dog–replica	−0.74	0.34	229	−2.20	0.038	−0.33	0.15	41	−0.64	−0.03
	plant–replica	−1.53	0.34	229	−4.56	<0.001	−0.69	0.15	41	−1.00	−0.38
Fp2	dog–plant	1.10	0.39	229	2.79	0.011	0.42	0.15	44	0.12	0.73
	plant–replica	−1.38	0.39	229	−3.51	0.002	−0.53	0.15	44	−0.84	−0.22
Mid beta											
Fp1	dog–plant	0.97	0.34	229	2.89	0.008	0.44	0.15	41	0.13	0.75
	plant–replica	−1.49	0.34	229	−4.42	<0.001	−0.67	0.15	41	−0.98	−0.36
Fp2	dog–plant	1.32	0.39	229	3.42	0.002	0.52	0.15	44	0.21	0.83
	plant–replica	−1.29	0.39	229	−3.34	0.002	−0.51	0.15	44	−0.82	−0.20
F3	dog–plant	0.80	0.32	229	2.50	0.040	0.38	0.15	45	0.07	0.69
Theta											
Fp1	dog–plant	0.84	0.41	229	2.04	0.042	0.31	0.15	42	0.00	0.62
	dog–replica	−1.09	0.41	229	−2.64	0.018	−0.40	0.15	42	−0.71	−0.09
	plant–replica	−1.93	0.41	229	−4.68	<0.001	−0.71	0.15	42	−1.02	−0.40
Fp2	plant–replica	−1.62	0.43	229	−3.73	0.001	−0.57	0.15	43	−0.87	−0.26
O1	dog–plant	−1.22	0.29	229	−4.16	<0.001	−0.63	0.15	41	−0.94	−0.32
	dog–replica	−1.03	0.29	229	−3.51	0.001	−0.53	0.15	41	−0.84	−0.22
O2	dog–plant	−1.31	0.27	224	−4.90	<0.001	−0.75	0.16	36	−1.07	−0.43
	dog–replica	−1.08	0.27	224	−4.03	<0.001	−0.62	0.16	36	−0.93	−0.30

SE = Standard error for parameters *x* and *y*; df = degree of freedom for parameters *x* and *y*; ES = effect size given by the Cohen’s *d*; CI = 95% confidence interval; *p* values < 0.05 were considered significant.

#### 3.2.1 Alpha

Significant differences in alpha power were observed across multiple electrodes. At Fp1, both the dog (*Mdiff* = −1.14, *SE* = 0.37, *t*(229) = −3.12, *p* = 0.004, *d* = −0.47, 95% CI [−0.78, −0.16]) and the plant (*Mdiff* = −1.35, *SE* = 0.37, *t*(229) = −3.70, *p* = 0.001, *d* = −0.56, 95% CI [−0.87, −0.25]) conditions showed lower alpha power compared to the replica condition. At Fp2, alpha power was lower in the plant condition than in the replica condition (*Mdiff* = −1.08, *SE* = 0.41, *t*(229) = −2.64, *p* = 0.026, *d* = −0.40, 95% CI [−0.71, −0.09]). At C3, alpha power was lower in the dog condition compared to the plant condition (*Mdiff* = −0.90, *SE* = 0.34, *t*(226) = −2.66, *p* = 0.025, *d* = −0.41, 95% CI [−0.71, −0.10]). Similarly, at C4, lower alpha power was observed in the dog condition compared to the plant (*Mdiff* = −0.69, *SE* = 0.28, *t*(229) = −2.46, *p* = 0.043, *d* = −0.37, 95% CI [−0.68, −0.07]). At P3, alpha power was reduced in the dog condition relative to the plant condition (*Mdiff* = −1.09, *SE* = 0.31, *t*(228) = −2.28, *p* = 0.002, *d* = −0.53, 95% CI [−0.84, −0.22]) and the replica condition (*Mdiff* = −0.83, *SE* = 0.35, *t*(228) = −2.66, *p* = 0.017, *d* = −0.40, 95% CI [−0.71, −0.09]). In posterior electrode O1, alpha power was lower in the dog condition than in both plant (*Mdiff* = −1.45, *SE* = 0.34, *t*(229) = −4.15, *p* < 0.001, *d* = −0.63, 95% CI [−0.94, −0.32]) and replica (*Mdiff* = −0.94, *SE* = 0.35, *t*(229) = −2.70, *p* = 0.015, *d* = −0.41, 95% CI [−0.72, −0.10]). At O2, the power in dog condition compared to the plant was significantly lower (*Mdiff* = −1.24, *SE* = 0.34, *t*(224) = −3.62, *p* = 0.001, *d* = −0.56, 95% CI [−0.87, −0.24]). Note that the difference between the replica and the dog was close to being significant for O2 (*Mdiff* = −0.75, *SE* = 0.34, *t*(224) = −2.18, *p* = 0.059, *d* = −0.33, 95% CI [−0.65, −0.02]).

#### 3.2.2 Slow alpha

The results in the slow alpha range showed a similar pattern. At Fp1, both the dog (*Mdiff* = −1.28, *SE* = 0.38, *t*(229) = −3.39, *p* = 0.002, *d* = −0.51, 95% CI [−0.82, −0.20]) and the plant (*Mdiff* = −1.32, *SE* = 0.38, *t*(229) = −3.49, *p* = 0.002, *d* = −0.53, 95% CI [−0.84, −0.22]) conditions had lower power than the replica. At Fp2, a difference was observed between the plant and the replica (*Mdiff* = −1.02, *SE* = 0.42, *t*(229) = −2.45, *p* = 0.045, *d* = −0.37, 95% CI [−0.68, −0.06]). A significant reduction in slow alpha power was also observed at F4 during the dog condition compared to the plant (*Mdiff* = −0.77, *SE* = 0.32, *t*(229) = −2.43, *p* = 0.047, *d* = −0.37, 95% CI [−0.68, −0.06]). At C3, alpha power was lower in the dog condition compared to both the plant (*Mdiff* = −1.09, *SE* = 0.34, *t*(226) = −3.20, *p* = 0.005, *d* = −0.49, 95% CI [−0.80, −0.18]) and the replica (*Mdiff* = −0.92, *SE* = 0.34, *t*(226) = −2.68, *p* = 0.016, *d* = −0.41, 95% CI [−0.72, −0.10]). The second central electrode, C4, also showed reduced slow alpha during the dog condition compared to the plant (*Mdiff* = −098, *SE* = 0.28, *t*(229) = −3.47, *p* = 0.002, *d* = −0.53, 95% CI [−0.84, −0.22]) and the replica (*Mdiff* = −0.66, *SE* = 0.28, *t*(229) = −2.35, *p* = 0.039, *d* = −0.36, 95% CI [−0.66, −0.05]). At P3, slow alpha power was reduced in the dog condition relative to the plant (*Mdiff* = −1.31, *SE* = 0.32, *t*(228) = −4.05, *p* < 0.001, *d* = −0.61, 95% CI [−0.93, −0.30]) and also relative to the replica (*Mdiff* = −0.98, *SE* = 0.32, *t*(228) = −3.02, *p* = 0.006, *d* = −0.46, 95% CI [−0.77, −0.15]). At P4, the power of slow alpha was reduced in the dog condition compared to the plant (*Mdiff* = −1.05, *SE* = 0.37, *t*(228) = −2.84, *p* = 0.015, *d* = −0.43, 95% CI [−0.74, −0.12]). Posterior electrodes O1 and O2 showed the largest differences, with lower power in the dog condition compared to the plant at O1 (*Mdiff* = −1.87, *SE* = 0.34, *t*(229) = −5.41, *p* < 0.001, *d* = −0.82, 95% CI [−1.13, −0.51]) and O2 (*Mdiff* = −1.65, *SE* = 0.35, *t*(224) = −4.75, *p* < 0.001, *d* = −0.73, 95% CI [−1.04, −0.42]). Similarly, the power was less important in the dog compared to the replica for O1 (*Mdiff* = −1.19, *SE* = 0.34, *t*(229) = −3.44, *p* = 0.001, *d* = −0.52, 95% CI [−0.83, −0.21]) and O2 (*Mdiff* = −1.02, *SE* = 0.35, *t*(224) = −2.93, *p* = 0.007, *d* = −0.45, 95% CI [−0.76, −0.14]). Exposure to the plant condition led to a tendentially greater power compared to the replica (*Mdiff* = 0.68, *SE* = 0.34, *t*(229) = 1.97, *p* = 0.050, *d* = 0.30, 95% CI [−0.01, 0.61]).

#### 3.2.3 Fast alpha

At Fp1, fast alpha power was lower in both the dog (*Mdiff* = −0.82, *SE* = 0.35, *t*(229) = −2.37, *p* = 0.037, *d* = −0.36, 95% CI [−0.67, −0.05]) and the plant (*Mdiff* = −1.44, *SE* = 0.35, *t*(229) = −4.17, *p* < 0.001, *d* = −0.63, 95% CI [−0.94, −0.32]) conditions compared to the replica condition. At Fp2, a reduction in power was also observed in the plant condition relative to the replica (*Mdiff* = −1.28, *SE* = 0.35, *t*(229) = −3.21, *p* = 0.005, *d* = −0.49, 95% CI [−0.79, −0.18]).

#### 3.2.4 Beta

In the beta band, power at Fp1 was higher in the dog condition compared to the plant (*Mdiff* = 1.13, *SE* = 0.34, *t*(229) = 3.30, *p* = 0.002, *d =* 0.50, 95% CI [0.19, 0.81]). Additionally, for the same electrode, power was lower in the plant condition compared to the replica (*Mdiff* = −1.45, *SE* = 0.34, *t*(229) = −4.26, *p* < 0.001, *d* = −0.65, 95% CI [−0.96, −0.33]). Fp2 had a similar pattern with a higher beta power in the dog condition compared to the plant (*Mdiff* = 1.49, *SE* = 0.39, *t*(229) = 3.81, *p* = 0.001, *d =* 0.58, 95% CI [0.27, 0.89]) and a reduced power in the plant condition compared to the replica (*Mdiff* = −1.33, *SE* = 0.39, *t*(229) = −3.40, *p* = 0.002, *d* = −0.52, 95% CI [−0.82, −0.21]). At frontal electrode F3, the beta power was higher in the dog condition compared to both the plant (*Mdiff* = 0.98, *SE* = 0.33, *t*(229) = 2.94, *p* = 0.011, *d =* 0.45, 95% CI [0.14, 0.75]) and the replica (*Mdiff* = 0.85, *SE* = 0.33, *t*(229) = 2.53, *p* = 0.024, *d =* 0.38, 95% CI [0.08, 0.69]). At C4, beta power was higher in the dog condition than in both the plant (*Mdiff* = 1.09, *SE* = 0.34, *t*(229) = 3.24, *p* = 0.004, *d =* 0.49, 95% CI [0.18, 0.80]) and the replica (*Mdiff* = 0.89, *SE* = 0.34, *t*(229) = 2.63, *p* = 0.018, *d =* 0.40, 95% CI [0.09, 0.71]) conditions.

#### 3.2.5 Low beta

At Fp1, power was lower in the dog (*Mdiff* = −0.74, *SE* = 0.34, *t*(229) = −2.20, *p* = 0.038, *d* = −0.33, 95% CI [−0.64, −0.03]) and the plant (*Mdiff* = −1.53, *SE* = 0.34, *t*(229) = −4.56, *p* < 0.001, *d* = −0.69, 95% CI [−1.00, −0.38]) conditions compared to the replica. For the same electrode, low beta power was greater during interaction with the dog compared to the plant (*Mdiff* = 0.79, *SE* = 0.34, *t*(229) = 2.36, *p* = 0.038, *d =* 0.36, 95% CI [0.05, 0.67]). At Fp2, power was higher in the dog condition compared to the plant (*Mdiff* = 1.10, *SE* = 0.39, *t*(229) = 2.79, *p* = 0.011, *d =* 0.42, 95% CI [0.12, 0.73]), and lower in the plant compared to the replica (*Mdiff* = −1.38, *SE* = 0.39, *t*(229) = −3.51, *p* = 0.002, *d* = −0.53, 95% CI [−0.84, −0.22]).

#### 3.2.6 Mid beta

At Fp1, mid beta power was higher in the dog condition compared to the plant (*Mdiff* = 0.97, *SE* = 0.34, *t*(229) = 2.89, *p* = 0.008, *d =* 0.44, 95% CI [0.13, 0.75]), and lower in the plant compared to the replica (*Mdiff* = −1.49, *SE* = 0.34, *t*(229) = −4.42, *p* < 0.001, *d* = −0.67, 95% CI [−0.98, −0.36]). At Fp2, power was higher in the dog condition compared to the plant (*Mdiff* = 1.32, *SE* = 0.39, *t*(229) = 3.42, *p* = 0.002, *d =* 0.52, 95% CI [0.21, 0.83]), and lower in the plant compared to the replica (*Mdiff* = −1.29, *SE* = 0.39, *t*(229) = −3.34, *p* = 0.002, *d* = −0.51, 95% CI [−0.82, −0.20]). At F3, power was also higher in the dog condition compared to the plant (*Mdiff* = 0.80, *SE* = 0.32, *t*(229) = 2.50, *p* = 0.040, *d =* 0.38, 95% CI [0.07, 0.69]).

#### 3.2.7 Theta

Theta power at Fp1 was lower in both the dog (*Mdiff* = −1.09, *SE* = 0.41, *t*(229) = −2.64, *p* = 0.018, *d* = −0.40, 95% CI [−0.71, −0.09]) and the plant (*Mdiff* = −1.93, *SE* = 0.41, *t*(229) = −4.68, *p* < 0.001, *d* = −0.71, 95% CI [−1.02, −0.40]) conditions compared to the replica condition. The theta power at Fp1 during the dog condition was greater than during the plant condition (*Mdiff* = 0.84, *SE* = 0.41, *t*(229) = 2.04, *p* = 0.042, *d =* 0.31, 95% CI [0.00, 0.62]). At Fp2, power was reduced in the plant condition relative to the replica (*Mdiff* = −1.62, *SE* = 0.43, *t*(229) = −3.73, *p* = 0.001, *d* = −0.57, 95% CI [−0.87, −0.26]). In the posterior regions, power was lower in the dog condition compared to both the plant at O1 (*Mdiff* = −1.22, *SE* = 0.29, *t*(229) = −4.16, *p* < 0.001, *d* = −0.63, 95% CI [−0.94, −0.32]) and the replica at O1 (*Mdiff* = −1.03, *SE* = 0.27, *t*(229) = −3.51, *p* = 0.001, *d* = −0.53, 95% CI [−0.84, −0.22]). Similar observations were made at O2 with a reduced theta power in the dog condition compared to the plant (*Mdiff* = −1.31, *SE* = 0.27, *t*(224) = −4.90, *p* < 0.001, *d* = −0.75, 95% CI [−1.07, −0.43]) and the replica (*Mdiff* = −1.08, *SE* = 0.27, *t*(224) = −4.03, *p* < 0.001, *d* = −0.62, 95% CI [−0.93, −0.30]).

## 4 Discussion

The present study investigated cortical dynamics of healthy human participants across three experimental conditions—interaction with a real dog, a plant, and a realistic dog replica—by exploratively analyzing EEG activity across multiple frequency bands. The LMMs and pairwise comparisons collectively revealed condition-specific patterns, with particularly distinct activity in the dog condition across alpha, beta, and theta bands. Participants had mostly lower alpha power in the dog condition compared to one or both control conditions, especially in the occipital, parietal, central, and prefrontal regions. Higher beta power was measured in participants in the frontal regions during the dog interaction, mostly in comparison with the plant condition. Participants exhibited lower theta power in various brain regions in the dog condition compared to one or both control conditions.

The observed decrease in alpha activity in the dog condition suggests heightened cortical engagement and attention. Reduced alpha power, particularly in the posterior and central regions, has been associated with increased visual attention and information-processing demands (Klimesch, 1999; Smith and Gevins, 2004; Lee and Lee, 2024). This interpretation has been supported by the identification of pronounced effects in occipital (O1, O2) and parietal (P3, P4) channels—regions implicated in sensory integration and visual–spatial awareness (Acuna, 2002; Gola et al., 2013). This is further supported by the observation of the suppression of the slow alpha band in the dog condition, which would confirm greater mobilization of attentional resources allocated to stimulus processing (Klimesch et al., 1997). Our findings suggest that interacting with a real dog, even under controlled experimental conditions, may activate mechanisms of attentional orientation and sensory monitoring to a greater extent than engagement with a replica dog or a plant.

Interestingly, the directionality of alpha changes was not the same as observed in other similar studies. Indeed, Teo et al. (2024) measured an increased alpha power during interactions with dogs in comparison with a control. They interpreted those findings as a relaxing effect felt during HDI. However, those findings were obtained from a single frontopolar electrode, which makes the comparison with our findings difficult as we found the changes in alpha bands mainly in the occipital and parietal regions. The analysis of Yoo et al. (2024) also emphasized an increased alpha power in the frontal regions when participants were playing and walking with a dog. They also interpreted these findings as a state of relaxation. However, due to differences in the type of activity and the brain regions involved, it is difficult to compare these findings with ours.

The finding that both the plant and the replica conditions elicited relatively higher alpha power implies that participants were less engaged or required fewer attentional resources when interacting with a nonanimal stimulus. This aligns with prior evidence showing that animals, and especially companion animals, act as socially salient stimuli capable of capturing human attention more effectively than objects (New et al., 2007; Kujala, 2017). From a broader perspective, alpha modulation may reflect the attentional engagement underlying the often-reported subjective experience of increased presence and involvement when interacting with animals compared to objects (Carbone et al., 2025).

Interestingly, most of the condition-related variation observed across the alpha spectrum appears to originate from the slow alpha band. Slow alpha has been suggested to reflect general attentional and alertness processes, whereas fast alpha is more tightly linked to specific cognitive or executive functions that might not be as strongly engaged in naturalistic interaction contexts (Sanfim et al., 2012). In addition, alpha power suppression in the broader 8–13 Hz range has been extensively associated with increased attentional engagement to external stimuli, consistent with the idea that participants were more engaged or externally oriented during certain interaction conditions (Bacigalupo and Luck, 2022).

Complementary to the findings of alpha activity, beta activity was significantly increased in the frontal and central electrodes in the dog condition, particularly compared to the plant and sometimes the replica dog. Beta oscillations are associated with sustained attention, active cognitive processing, and sensorimotor readiness (Ray and Cole, 1985; Kropotov, 2009; Andreassi, 2010; Engel and Fries, 2010; Abhang et al., 2016). Elevated beta power in frontal areas (Fp1, Fp2, F3, F4) may reflect enhanced emotional and attentional regulation, while central activity (e.g., C4) likely indexes increased motor preparation or sensorimotor engagement (Wood and Grafman, 2003; Kropotov, 2009; Kolb et al., 2012; Calcaterra et al., 2015). These patterns support interpretations from previous HAI studies, which have shown increased beta activity during dog interactions (Teo et al., 2024; Yoo et al., 2024), underscoring the cognitively and emotionally engaging nature of real animal presence.

Sub-band analyses indicated that these effects were most robust in the broader beta range (13–30 Hz), with more spatially restricted effects in low and mid beta. Broad beta activity has been proposed to reflect sustained maintenance of the current cognitive–sensorimotor state, whereas low beta is more closely linked to embodied and sensorimotor processes and mid beta to top-down monitoring and cognitive control (Engel and Fries, 2010; Kilavik et al., 2013; Spitzer and Haegens, 2017). The persistence of low-beta effects in prefrontal regions, including a left prefrontal increase relative to the replica condition, may therefore indicate enhanced embodied engagement during interaction with the real dog.

Interestingly, the replica condition occasionally elicited stronger beta power than either the dog or the plant, particularly in low and mid beta ranges. This might suggest that the replica prompted evaluative or compensatory processing—participants may have attempted to attribute agency or meaning to an inanimate object resembling a dog. Such responses would be consistent with research showing that anthropomorphic or ambiguous stimuli can engage cognitive networks differently than clearly animate or inanimate entities (Krach et al., 2008; Urquiza-Haas and Kotrschal, 2015).

Theta activity also distinguished the dog condition from the controls. In the occipital regions (O1, O2), theta power was lower during the dog condition compared to the control conditions. This reduction in posterior theta may reflect a state of increased external attentional focus and perceptual engagement. Theta oscillations in occipital areas have been associated with internally oriented processes such as visual imagery, emotional memory, or mind wandering (Lomas et al., 2015). Their reduction may thus suggest a shift away from internally directed cognition toward more externally driven sensory processing during interaction with a real dog. In contrast, increased theta power in the frontal and central regions during a replica condition might indicate reduced cognitive engagement or a shift toward internally focused processing, consistent with lower arousal or task disengagement (Cavanagh and Frank, 2014). These topographical differences underscore how the perceived social salience, emotional engagement, and realism of stimuli—such as a living dog versus an artificial replica—can differentially modulate brain dynamics associated with attention, emotion, and cognition across the cortical regions.

Session effects, which indicate significant changes from one session to another, were observed predominantly in the frontal areas, which warrants attention. Across 70 tested LMMs (10 electrodes × 7 frequency bands), session effects were significant in 23 models (32.9%). These effects clustered predominantly at the frontal and central sites (Fp1, Fp2, F3, C3), with fewer occurrences at the parietal or central-posterior sites (P4, C4). However, the direction and cause of these effects remain inconclusive. While they could reflect learning, habituation, or fatigue, a similar study (Rejer et al., 2022) failed to confirm robust habituation trends in EEG under repeated exposure. The current findings suggest subtle, systematic modulation of cortical activity over time, which deserves further exploration in longitudinal HAI paradigms.

Taken together, our results suggest that dog interaction is characterized by heightened attentional engagement (alpha suppression), emotional arousal (theta suppression), and sensorimotor engagement and cognitive processing (beta modulation). This constellation of oscillatory changes aligns with behavioral and physiological studies reporting increased attention, reduced stress markers, and heightened emotional engagement during human–dog interaction (Beetz et al., 2012; Payne et al., 2015). The present findings extend this literature by providing neural evidence of these processes at the level of oscillatory dynamics, thus bridging subjective and physiological accounts of human–animal interaction. The results support the biophilia hypothesis (Wilson, 1986), which posits an innate human affinity for other living organisms, and align with growing evidence from HAI research indicating physiological, psychological, and cognitive benefits of animal presence, including reduced stress, enhanced emotional responsiveness, and increased attentional control (McConnell et al., 2011; Gee et al., 2021). While previous research has reported increased alpha activity during relaxed dog interactions (Teo et al., 2024; Yoo et al., 2024), our study emphasizes the cognitively activating and socially engaging aspects of direct, structured interaction with a live animal. This contrast with previous observations highlights the complexity of HAI outcomes and the importance of context, individual differences, and interaction modality in shaping neural and behavioral responses. Moreover, the results highlight the specificity of the dog condition compared to both the plant and the replica controls. While plant interaction may control for aspects of tactile stimulation and naturalness, and replica interaction for visual form, tactile stimuli, and maybe even cuteness, neither reproduced the neural signature elicited by a living animal. This underscores the importance of considering agency and reciprocity when investigating the mechanisms of HAI.

### 4.1 Strengths, limitations, and future directions

This study’s strengths included its within-subject design, allowing repeated measurements in the same individuals to improve reliability and reduce variability from external factors, which is key in neurocognitive research. The sample size was relatively large, with 428 usable EEG recordings, and the sessions were randomized to reduce habituation and to control order effects. The intervention was carefully controlled to isolate the effects of interacting with a living being, and the standardized setup minimized confounding factors like time-of-day effects. Additionally, all the conditions included stroking movements, ensuring that EEG differences were not due to motor activity alone.

Our study had some limitations. First, due to the nature of the intervention, blinding was not possible for either the experimenter or the participants. The selection of subjects for the study was also a limitation as only people who declared no fear of or allergies to dogs could be included, which might not be representative of the general population. In addition, although the recruitment was open without an age limitation, the majority of participants were students, which again limits the representativity of the sample. Concerning the dogs, while their breeds were different, their shape and size were comparable. Not including small dogs limits the representativity. Finally, the numerous comparisons done between each condition for all the electrodes and frequencies leads us to interpret the results with caution. It is important to note that the analyses presented here were an explorative and not predefined part of a preregistered study.

Future studies should replicate our findings with a confirmatory study design. Also, they should include broader human and dog populations to increase the representativity of the sample as well as a greater diversity in control conditions to test the neurological effects of HDI in real-life or therapeutic contexts.

Furthermore, it will be important to explore whether these neural signatures translate into behavioral or psychological outcomes, such as enhanced learning, stress reduction, or prosocial behavior. Longitudinal studies and real-world applications (e.g., in therapeutic, educational, or occupational settings) would help elucidate the functional significance of the observed effects.

### 4.2 Conclusion

In summary, the present study demonstrates that interacting with a dog induces distinct oscillatory patterns compared to interacting with a replica dog or a plant. Reduced alpha and theta power suggest heightened attentional engagement and arousal, while beta modulation points to sensorimotor and anticipatory processes. These results provide electrophysiological evidence that human–dog interaction is neurally distinct from interactions with nonanimal stimuli, emphasizing the social and emotional salience of companion animals. Beyond their contribution to HAI research, these findings have potential implications for applied contexts such as animal-assisted interventions, where understanding the neural mechanisms of engagement and emotional regulation could inform practice and optimize therapeutic outcomes.

## Data Availability

The datasets presented in this study can be found in online repositories. The names of the repository/repositories and accession number(s) can be found at: Open Science Framework repository.

## References

[ref1] Abhang P. A. Gawali B. W. Mehrotra S. C. (2016). “Technical aspects of brain rhythms and speech parameters” in Introduction to EEG- and speech-based emotion recognition (Amsterdam: Elsevier), 51–79. doi: 10.1016/B978-0-12-804490-2.00003-8

[ref2] Acuna B. D. (2002). Frontal and parietal lobe activation during transitive inference in humans. Cereb. Cortex 12, 1312–1321. doi: 10.1093/cercor/12.12.1312, 12427681

[ref3] Andreassi J. L. (2010). Psychophysiology. New York: Psychology Press. doi: 10.4324/9780203880340

[ref4] Bacigalupo F. Luck S. J. (2022). Alpha-band EEG suppression as a neural marker of sustained attentional engagement to conditioned threat stimuli. Soc. Cogn. Affect. Neurosci. 17, 1101–1117. doi: 10.1093/scan/nsac029, 35434733 PMC9766959

[ref5] Bao K. J. Schreer G. (2016). Pets and happiness: examining the association between pet ownership and wellbeing. Anthrozoös 29, 283–296. doi: 10.1080/08927936.2016.1152721

[ref6] Bates D. Mächler M. Bolker B. Walker S. (2015). Fitting linear mixed-effects models using lme4. J. Stat. Softw. 67, 1–48. doi: 10.18637/jss.v067.i01

[ref7] Beetz A. M. (2017). Theories and possible processes of action in animal assisted interventions. Appl. Dev. Sci. 21, 139–149. doi: 10.1080/10888691.2016.1262263

[ref8] Beetz A. Uvnäs-Moberg K. Julius H. Kotrschal K. (2012). Psychosocial and psychophysiological effects of human-animal interactions: the possible role of oxytocin. Front. Psychol. 3:234. doi: 10.3389/fpsyg.2012.00234, 22866043 PMC3408111

[ref9] Borgi M. Cirulli F. (2016). Pet face: mechanisms underlying human-animal relationships. Front. Psychol. 7:298. doi: 10.3389/fpsyg.2016.00298, 27014120 PMC4782005

[ref10] Calcaterra V. Veggiotti P. Palestrini C. De Giorgis V. Raschetti R. Tumminelli M. . (2015). Post-operative benefits of animal-assisted therapy in pediatric surgery: a randomised study. PLoS One 10:e0125813. doi: 10.1371/journal.pone.0125813, 26039494 PMC4454536

[ref11] Carbone F. Gerber E.-Y. Rérat C. Hattendorf J. Hediger K. (2025). Neuromechanisms and subjective experiences during human-dog interactions: assessing motivation and mental state in a randomized, controlled trial. PLoS One 20:e0325325. doi: 10.1371/journal.pone.0325325, 40460350 PMC12133184

[ref12] Cavanagh J. F. Frank M. J. (2014). Frontal theta as a mechanism for cognitive control. Trends Cogn. Sci. 18, 414–421. doi: 10.1016/j.tics.2014.04.012, 24835663 PMC4112145

[ref13] Chikhi S. Matton N. Blanchet S. (2022). EEG power spectral measures of cognitive workload: a meta-analysis. Psychophysiology 59:e14009. doi: 10.1111/psyp.14009, 35128686

[ref14] Cho S.-H. (2017). Effects of horseback riding exercise on the relative alpha power spectrum in the elderly. Arch. Gerontol. Geriatr. 70, 141–147. doi: 10.1016/j.archger.2017.01.011, 28135668

[ref15] Corcoran A. W. Alday P. M. Schlesewsky M. Bornkessel-Schlesewsky I. (2018). Toward a reliable, automated method of individual alpha frequency (IAF) quantification. Psychophysiology 55:e13064. doi: 10.1111/psyp.13064, 29357113

[ref16] Curl A. L. Bibbo J. Johnson R. A. (2021). Neighborhood engagement, dogs, and life satisfaction in older adulthood. J. Appl. Gerontol. 40, 1706–1714. doi: 10.1177/0733464820953725, 32909494

[ref17] Delorme A. (2021). eegstats. San Diego: Arnaud Delorme. Available online at: https://github.com/sccn/eegstats

[ref18] Delorme A. Makeig S. (2004). EEGLAB: an open source toolbox for analysis of single-trial EEG dynamics including independent component analysis. J. Neurosci. Methods 134, 9–21. doi: 10.1016/j.jneumeth.2003.10.009, 15102499

[ref19] Engel A. K. Fries P. (2010). Beta-band oscillations—signalling the status quo? Curr. Opin. Neurobiol. 20, 156–165. doi: 10.1016/j.conb.2010.02.015, 20359884

[ref20] Gandenberger J. Flynn E. Moratto E. Wendt A. Morris K. N. (2022). Molecular biomarkers of adult human and dog stress during canine-assisted interventions: a systematic scoping review. Animals 12:651. doi: 10.3390/ani12050651, 35268219 PMC8909518

[ref21] Gee N. R. Rodriguez K. E. Fine A. H. Trammell J. P. (2021). Dogs supporting human health and well-being: a biopsychosocial approach. Front. Vet. Sci. 8:630465. doi: 10.3389/fvets.2021.630465, 33860004 PMC8042315

[ref22] Gkintoni E. Halkiopoulos C. (2025). Mapping EEG metrics to human affective and cognitive models: an interdisciplinary scoping review from a cognitive neuroscience perspective. Biomimetics 10:730. doi: 10.3390/biomimetics10110730, 41294401 PMC12649996

[ref23] Gola M. Magnuski M. Szumska I. Wróbel A. (2013). EEG beta band activity is related to attention and attentional deficits in the visual performance of elderly subjects. Int. J. Psychophysiol. 89, 334–341. doi: 10.1016/j.ijpsycho.2013.05.007, 23688673

[ref24] Homma A. Hara H. Matsuzaki K. Sasaki M. Masaoka Y. Homma I. (2011). The effect of touching a dolphin on the EEG slow waves in children. Showa Univ. J. Med. Sci. 23, 115–119. doi: 10.15369/sujms.23.115

[ref25] Janssens M. Janssens E. Eshuis J. Lataster J. Simons M. Reijnders J. . (2021). Companion animals as buffer against the impact of stress on affect: an experience sampling study. Animals 11:2171. doi: 10.3390/ani11082171, 34438629 PMC8388427

[ref26] Jegatheesan B. Beetz A. Ormerod E. Johnson R. Fine A. Yamazaki K. . (2018). The IAHAIO definitions for animal assisted intervention and guidelines for wellness of animals involved in AAI. Available online at: https://iahaio.org/wp/wp-content/uploads/2018/04/iahaio_wp_updated-2018-final.pdf (Accessed January 15, 2024).

[ref27] Junça-Silva A. (2022). Friends with benefits: the positive consequences of pet-friendly practices for workers’ well-being. Int. J. Environ. Res. Public Health 19:1069. doi: 10.3390/ijerph19031069, 35162092 PMC8834589

[ref28] Kilavik B. E. Zaepffel M. Brovelli A. MacKay W. A. Riehle A. (2013). The ups and downs of beta oscillations in sensorimotor cortex. Exp. Neurol. 245, 15–26. doi: 10.1016/j.expneurol.2012.09.014, 23022918

[ref29] Klimesch W. (1999). EEG alpha and theta oscillations reflect cognitive and memory performance: a review and analysis. Brain Res. Rev. 29, 169–195. doi: 10.1016/S0165-0173(98)00056-3, 10209231

[ref30] Klimesch W. Doppelmayr M. Pachinger T. Russegger H. (1997). Event-related desynchronization in the alpha band and the processing of semantic information. Cogn. Brain Res. 6, 83–94. doi: 10.1016/S0926-6410(97)00018-9, 9450602

[ref31] Kogan L. R. Currin-McCulloch J. Bussolari C. Packman W. Erdman P. (2021). The psychosocial influence of companion animals on positive and negative affect during the COVID-19 pandemic. Animals 11:2084. doi: 10.3390/ani11072084, 34359212 PMC8300185

[ref32] Kolb B. Mychasiuk R. Muhammad A. Li Y. Frost D. O. Gibb R. (2012). Experience and the developing prefrontal cortex. Proc. Natl. Acad. Sci. 109, 17186–17193. doi: 10.1073/pnas.1121251109, 23045653 PMC3477383

[ref33] Krach S. Hegel F. Wrede B. Sagerer G. Binkofski F. Kircher T. (2008). Can machines think? Interaction and perspective taking with robots investigated via fMRI. PLoS One 3:e2597. doi: 10.1371/journal.pone.0002597, 18612463 PMC2440351

[ref34] Krause-Parello C. A. Gulick E. E. Basin B. (2019). Loneliness, depression, and physical activity in older adults: the therapeutic role of human–animal interactions. Anthrozoös 32, 239–254. doi: 10.1080/08927936.2019.1569906

[ref35] Kropotov J. D. (2009). “Beta rhythms” in Quantitative EEG, event-related potentials and neurotherapy (New York: Elsevier), 59–76. doi: 10.1016/B978-0-12-374512-5.00003-7

[ref36] Kujala M. V. (2017). Canine emotions as seen through human social cognition. Anim. Sentience 2, 1–34. doi: 10.51291/2377-7478.1114

[ref37] Lee H.-S. Lee J.-Y. (2024). Assessing the restorative effects of observing a video of dog play in urban dog parks using EEG. Hum. Animal Interact. 12. doi: 10.1079/hai.2024.0037

[ref38] Lenth R. V. Piaskowski J. (2017). Emmeans: estimated marginal means, aka least-squares means. CRAN: Contributed Packages. 34, 216–221. doi: 10.32614/CRAN.package.emmeans

[ref39] Levin A. R. Naples A. J. Scheffler A. W. Webb S. J. Shic F. Sugar C. A. . (2020). Day-to-day test-retest reliability of EEG profiles in children with autism Spectrum disorder and typical development. Front. Integr. Neurosci. 14:21. doi: 10.3389/fnint.2020.00021, 32425762 PMC7204836

[ref40] Liu H. Lin J. Lin W. (2024). Cognitive mechanisms and neurological foundations of companion animals’ role in enhancing human psychological well-being. Front. Psychol. 15:1354220. doi: 10.3389/fpsyg.2024.1354220, 38721326 PMC11076790

[ref41] Lomas T. Ivtzan I. Fu C. H. Y. (2015). A systematic review of the neurophysiology of mindfulness on EEG oscillations. Neurosci. Biobehav. Rev. 57, 401–410. doi: 10.1016/j.neubiorev.2015.09.018, 26441373

[ref42] McConnell A. R. Brown C. M. Shoda T. M. Stayton L. E. Martin C. E. (2011). Friends with benefits: on the positive consequences of pet ownership. J. Pers. Soc. Psychol. 101, 1239–1252. doi: 10.1037/a0024506, 21728449

[ref43] Mognon A. Jovicich J. Bruzzone L. Buiatti M. (2011). ADJUST: an automatic EEG artifact detector based on the joint use of spatial and temporal features. Psychophysiology 48, 229–240. doi: 10.1111/j.1469-8986.2010.01061.x, 20636297

[ref44] New J. Cosmides L. Tooby J. (2007). Category-specific attention for animals reflects ancestral priorities, not expertise. Proc. Natl. Acad. Sci. 104, 16598–16603. doi: 10.1073/pnas.0703913104, 17909181 PMC2034212

[ref45] Oh Y. A. Kim S. O. Park S. A. (2019). Real foliage plants as visual stimuli to improve concentration and attention in elementary students. Int. J. Environ. Res. Public Health 16:796. doi: 10.3390/ijerph16050796, 30841505 PMC6427160

[ref46] Payne E. Bennett P. McGreevy P. (2015). Current perspectives on attachment and bonding in the dog-human dyad. Psychol. Res. Behav. Manag. 8, 71–79. doi: 10.2147/PRBM.S74972, 25750549 PMC4348122

[ref47] Ray W. J. Cole H. W. (1985). EEG alpha activity reflects attentional demands, and beta activity reflects emotional and cognitive processes. Science 228, 750–752. doi: 10.1126/science.3992243, 3992243

[ref48] Rejer I. Wacewicz D. Schab M. Romanowski B. Łukasiewicz K. Maciaszczyk M. (2022). Stressors length and the habituation effect—an EEG study. Sensors 22:6862. doi: 10.3390/s22186862, 36146211 PMC9505843

[ref49] Sanfim A. Velasques B. Machado S. Arias-Carrión O. Paes F. Teixeira S. . (2012). Analysis of slow- and fast-alpha band asymmetry during performance of a saccadic eye movement task: dissociation between memory- and attention-driven systems. J. Neurol. Sci. 312, 62–67. doi: 10.1016/j.jns.2011.08.022, 21880332

[ref50] Schlogl A. Brunner C. (2008). BioSig: a free and open source software library for BCI research. Computer 41, 44–50. doi: 10.1109/MC.2008.407

[ref51] Smith M. E. Gevins A. (2004). Attention and brain activity while watching television: components of viewer engagement. Media Psychol. 6, 285–305. doi: 10.1207/s1532785xmep0603_3

[ref52] Spitzer B. Haegens S. (2017). Beyond the status quo: a role for beta oscillations in endogenous content (re)activation. eNeuro 4:ENEURO.0170-17.2017. doi: 10.1523/ENEURO.0170-17.2017, 28785729 PMC5539431

[ref53] Teo J. T. Johnstone S. J. Römer S. S. Thomas S. J. (2022). Psychophysiological mechanisms underlying the potential health benefits of human-dog interactions: a systematic literature review. Int. J. Psychophysiol. 180, 27–48. doi: 10.1016/j.ijpsycho.2022.07.007, 35901904

[ref54] Teo J. T. Johnstone S. J. Thomas S. J. (2024). Brain and heart activity during interactions with pet dogs: a portable electroencephalogram and heart rate variability study. Int. J. Psychophysiol. 204:112412. doi: 10.1016/j.ijpsycho.2024.112412, 39111638

[ref55] Urquiza-Haas E. G. Kotrschal K. (2015). The mind behind anthropomorphic thinking: attribution of mental states to other species. Anim. Behav. 109, 167–176. doi: 10.1016/j.anbehav.2015.08.011

[ref56] Wilson E. O. (1986). Biophilia. Cambridge: Harvard University Press. Available at: http://www.jstor.org/stable/10.2307/j.ctvk12s6h

[ref57] Wood J. N. Grafman J. (2003). Human prefrontal cortex: processing and representational perspectives. Nat. Rev. Neurosci. 4, 139–147. doi: 10.1038/nrn1033, 12563285

[ref58] World Medical Association (2013). World medical association declaration of Helsinki: ethical principles for medical research involving human subjects. JAMA 310, 2191–2194. doi: 10.1001/jama.2013.281053 24141714

[ref59] Xu X. Shen X. Chen X. Zhang Q. Wang S. Li Y. . (2025). A multi-context emotional EEG dataset for cross-context emotion decoding. Sci Data 12:1142. doi: 10.1038/s41597-025-05349-2, 40610470 PMC12229444

[ref60] Xu G. Wang Z. Zhao X. Li R. Zhou T. Xu T. . (2024). A subject-specific attention index based on the weighted spectral power. IEEE Trans. Neural Syst. Rehabil. Eng. 32, 1687–1702. doi: 10.1109/TNSRE.2024.3392242, 38648157

[ref61] Yoo O. Wu Y. Han J. S. Park S.-A. (2024). Psychophysiological and emotional effects of human–dog interactions by activity type: an electroencephalogram study. PLoS One 19:e0298384. doi: 10.1371/journal.pone.0298384, 38478472 PMC10936815

