# Supplementary material for: Neural signature of human–dog interactions: EEG correlates from comparisons with plant and replica dog in a within-subject, cross-over randomized trial
Source: Front Hum Neurosci. 2026 Feb 6;20:1731796. doi: 10.3389/fnhum.2026.1731796 (PMC12920550; doi:10.3389/fnhum.2026.1731796)
Supplement: Supplementary file 1 [file Data_Sheet_1.docx]

Supplementary Material

Figure S1.


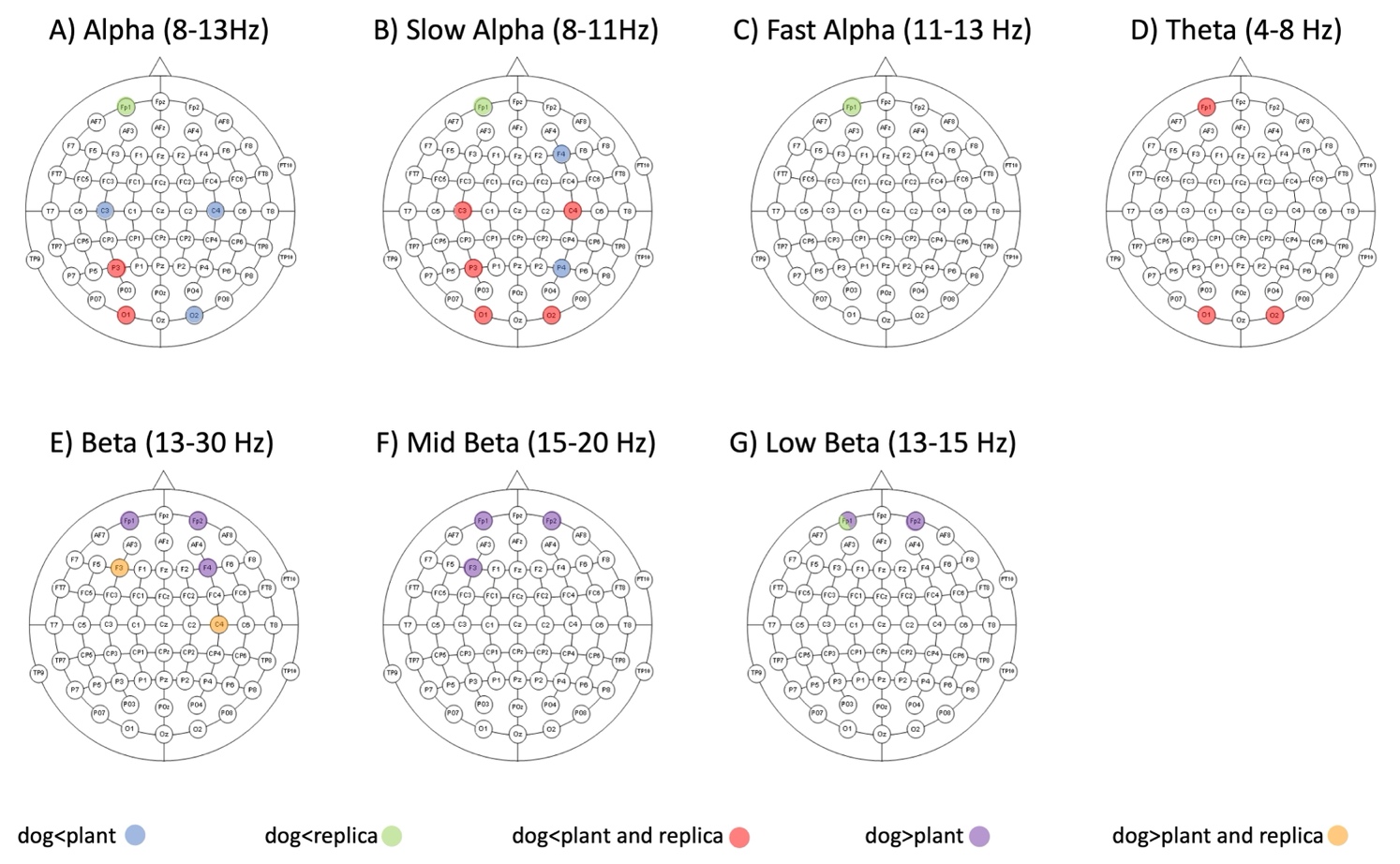


**Figure S1.** **Graphical representation of significant pairwise comparisons.** Significant pairwise comparisons (p<0.05) showing directionality of power differences for the dog condition vs the controls are reported with a color code at each electrode location and for each frequency. (A) alpha (8–13Hz); (B) slow alpha (8–11Hz); (C) fast alpha (11–13Hz); (D) theta (4–8Hz); (E) beta (13–30Hz); (F) mid beta (15–20Hz); (G) low beta (13–15Hz). Blue=dog<plant; green=dog<replica; red=dog<plant and replica; purple=dog>plant; orange=dog>plant and replica. Note that only electrodes Fp1/2, F3/4, C3/4, P3/4 and O1/2 were included in the analysis. 64 channels EEG scheme from https://www.fieldtriptoolbox.org/assets/img/faq/capmapping/easycapm11.png

Table S1.

|  | Alpha | | | | | | | | | |
| --- | --- | --- | --- | --- | --- | --- | --- | --- | --- | --- |
|  | B1 | | Dog | | Plant | | Replica | | B2 | |
|  | Mean | SD | Mean | SD | Mean | SD | Mean | SD | Mean | SD |
| Fp1 | -2.16 | 4.31 | -1.92 | 3.92 | -2.13 | 2.49 | -0.77 | 3.37 | -1.48 | 4.44 |
| Fp2 | -1.84 | 4.02 | -1.71 | 4.09 | -2.04 | 2.64 | -0.96 | 3.77 | -1.23 | 4.31 |
| F3 | -2.11 | 3.33 | -3.97 | 2.98 | -3.64 | 2.74 | -3.75 | 2.79 | -1.37 | 3.45 |
| F4 | -1.67 | 3.28 | -3.43 | 3.34 | -2.86 | 2.82 | -2.91 | 2.84 | -1.19 | 3.34 |
| C3 | -3.21 | 3.38 | -6.23 | 2.74 | -5.33 | 2.92 | -5.50 | 2.60 | -2.41 | 3.09 |
| C4 | -2.67 | 3.38 | -5.78 | 2.71 | -5.09 | 2.40 | -5.38 | 2.48 | -2.41 | 3.12 |
| P3 | -1.15 | 4.21 | -4.92 | 2.97 | -3.84 | 3.37 | -4.13 | 2.89 | -0.21 | 4.01 |
| P4 | -1.26 | 4.32 | -4.48 | 3.40 | -3.67 | 3.44 | -4.11 | 3.44 | 0.11 | 3.76 |
| O1 | 2.13 | 4.48 | -0.80 | 3.86 | 0.65 | 3.78 | 0.15 | 3.43 | 2.86 | 4.51 |
| O2 | 2.20 | 4.37 | -0.75 | 3.64 | 0.52 | 3.89 | 0.02 | 3.45 | 3.02 | 4.37 |
| Slow Alpha | | | | | | | | | | |
|  | B1 | | Dog | | Plant | | Replica | | B2 | |
|  | Mean | SD | Mean | SD | Mean | SD | Mean | SD | Mean | SD |
| Fp1 | -1.66 | 4.49 | -1.49 | 3.99 | -1.53 | 2.66 | -0.21 | 3.44 | -0.78 | 4.64 |
| Fp2 | -1.29 | 4.14 | -1.31 | 4.13 | -1.43 | 2.78 | -0.41 | 3.77 | -0.57 | 4.52 |
| F3 | -1.54 | 3.55 | -3.51 | 2.95 | -2.97 | 2.92 | -3.16 | 2.86 | -0.60 | 3.70 |
| F4 | -1.12 | 3.51 | -2.98 | 3.35 | -2.21 | 2.97 | -2.32 | 2.87 | -0.47 | 3.62 |
| C3 | -2.84 | 3.53 | -5.91 | 2.68 | -4.82 | 2.98 | -5.00 | 2.63 | -1.87 | 3.32 |
| C4 | -2.39 | 3.48 | -5.54 | 2.65 | -4.56 | 2.49 | -4.87 | 2.49 | -1.90 | 3.39 |
| P3 | -0.73 | 4.29 | -4.60 | 2.97 | -3.29 | 3.52 | -3.65 | 2.95 | 0.42 | 4.27 |
| P4 | -0.83 | 4.62 | -4.15 | 3.45 | -3.11 | 3.61 | -3.61 | 3.54 | 0.79 | 4.12 |
| O1 | 2.60 | 4.68 | -0.57 | 3.76 | 1.30 | 3.97 | 0.62 | 3.46 | 3.52 | 4.75 |
| O2 | 2.71 | 4.61 | -0.50 | 3.57 | 1.17 | 4.02 | 0.54 | 3.52 | 3.74 | 4.59 |
| Fast Alpha | | | | | | | | | | |
|  | B1 | | Dog | | Plant | | Replica | | B2 | |
|  | Mean | SD | Mean | SD | Mean | SD | Mean | SD | Mean | SD |
| Fp1 | -3.27 | 4.17 | -2.67 | 3.82 | -3.29 | 2.17 | -1.85 | 3.28 | -3.16 | 4.09 |
| Fp2 | -3.03 | 4.03 | -2.41 | 4.05 | -3.27 | 2.42 | -1.99 | 3.80 | -2.82 | 4.01 |
| F3 | -3.37 | 3.16 | -4.77 | 3.19 | -5.05 | 2.41 | -4.91 | 2.73 | -3.21 | 3.19 |
| F4 | -2.90 | 3.13 | -4.22 | 3.46 | -4.30 | 2.67 | -4.11 | 2.92 | -2.96 | 3.06 |
| C3 | -3.98 | 3.67 | -6.75 | 2.95 | -6.32 | 3.05 | -6.38 | 2.73 | -3.60 | 3.31 |
| C4 | -3.35 | 3.74 | -6.16 | 2.99 | -6.15 | 2.48 | -6.29 | 2.63 | -3.59 | 3.21 |
| P3 | -2.10 | 4.56 | -5.48 | 3.15 | -4.95 | 3.17 | -5.04 | 2.89 | -1.79 | 3.92 |
| P4 | -2.36 | 4.10 | -4.99 | 3.48 | -4.78 | 3.14 | -5.06 | 3.40 | -1.54 | 3.55 |
| O1 | 1.04 | 4.19 | -1.27 | 4.08 | -0.73 | 3.39 | -0.81 | 3.43 | 1.37 | 4.11 |
| O2 | 1.11 | 4.15 | -1.17 | 3.91 | -0.79 | 3.74 | -0.95 | 3.43 | 1.47 | 4.22 |
| Beta | | | | | | | | | | |
|  | B1 | | Dog | | Plant | | Replica | | B2 | |
|  | Mean | SD | Mean | SD | Mean | SD | Mean | SD | Mean | SD |
| Fp1 | -6.21 | 3.87 | -5.01 | 3.86 | -6.13 | 2.17 | -4.68 | 3.11 | -6.14 | 3.77 |
| Fp2 | -5.77 | 3.70 | -4.65 | 3.91 | -6.14 | 2.42 | -4.81 | 3.57 | -5.28 | 3.73 |
| F3 | -6.91 | 3.03 | -6.91 | 3.52 | -7.89 | 2.30 | -7.75 | 2.59 | -6.82 | 2.82 |
| F4 | -6.16 | 3.19 | -6.39 | 3.70 | -7.34 | 2.56 | -7.10 | 2.98 | -6.17 | 3.09 |
| C3 | -8.63 | 2.63 | -9.10 | 3.07 | -9.76 | 2.74 | -9.38 | 2.78 | -8.25 | 2.71 |
| C4 | -8.30 | 2.41 | -8.59 | 3.50 | -9.68 | 2.06 | -9.48 | 2.67 | -8.56 | 2.38 |
| P3 | -7.88 | 3.18 | -8.76 | 2.53 | -9.11 | 2.43 | -8.98 | 2.22 | -7.66 | 2.58 |
| P4 | -8.25 | 3.09 | -8.50 | 3.27 | -9.09 | 2.51 | -9.13 | 2.85 | -7.71 | 2.52 |
| O1 | -4.99 | 2.97 | -5.29 | 3.14 | -5.67 | 2.42 | -5.70 | 2.52 | -4.73 | 2.81 |
| O2 | -5.08 | 3.05 | -5.55 | 3.06 | -6.04 | 2.74 | -5.95 | 2.85 | -4.94 | 2.75 |
| Mid Beta | | | | | | | | | | |
|  | B1 | | Dog | | Plant | | Replica | | B2 | |
|  | Mean | SD | Mean | SD | Mean | SD | Mean | SD | Mean | SD |
| Fp1 | -5.82 | 3.75 | -4.59 | 3.79 | -5.56 | 2.10 | -4.08 | 3.17 | -5.76 | 3.51 |
| Fp2 | -5.50 | 3.56 | -4.22 | 3.89 | -5.55 | 2.39 | -4.25 | 3.59 | -5.03 | 3.32 |
| F3 | -6.33 | 2.93 | -6.46 | 3.38 | -7.26 | 2.22 | -7.13 | 2.54 | -6.18 | 2.73 |
| F4 | -5.56 | 3.13 | -5.99 | 3.58 | -6.68 | 2.56 | -6.43 | 2.93 | -5.53 | 2.96 |
| C3 | -7.96 | 2.67 | -8.69 | 2.89 | -9.07 | 2.67 | -8.69 | 2.76 | -7.39 | 2.74 |
| C4 | -7.53 | 2.40 | -8.27 | 3.11 | -8.90 | 1.93 | -8.70 | 2.55 | -7.61 | 2.42 |
| P3 | -6.79 | 3.27 | -8.12 | 2.52 | -8.12 | 2.41 | -8.03 | 2.24 | -6.39 | 2.73 |
| P4 | -7.21 | 3.19 | -7.86 | 3.20 | -8.12 | 2.51 | -8.18 | 2.87 | -6.49 | 2.63 |
| O1 | -3.76 | 3.14 | -4.64 | 3.03 | -4.58 | 2.47 | -4.67 | 2.54 | -3.51 | 2.96 |
| O2 | -3.84 | 3.16 | -4.86 | 2.94 | -4.92 | 2.82 | -4.91 | 2.94 | -3.62 | 2.81 |
| Low Beta | | | | | | | | | | |
|  | B1 | | Dog | | Plant | | Replica | | B2 | |
|  | Mean | SD | Mean | SD | Mean | SD | Mean | SD | Mean | SD |
| Fp1 | -4.77 | 3.73 | -3.59 | 3.79 | -4.38 | 1.98 | -2.85 | 3.24 | -4.87 | 3.59 |
| Fp2 | -4.54 | 3.62 | -3.28 | 4.02 | -4.38 | 2.26 | -3.00 | 3.72 | -4.35 | 3.48 |
| F3 | -5.16 | 2.72 | -5.66 | 3.08 | -6.21 | 2.08 | -6.02 | 2.57 | -5.14 | 2.69 |
| F4 | -4.55 | 2.81 | -5.16 | 3.38 | -5.56 | 2.49 | -5.22 | 2.88 | -4.74 | 2.64 |
| C3 | -6.11 | 2.86 | -7.71 | 2.79 | -7.61 | 2.73 | -7.54 | 2.61 | -5.85 | 2.73 |
| C4 | -5.53 | 2.91 | -7.26 | 2.82 | -7.49 | 2.10 | -7.48 | 2.45 | -5.88 | 2.65 |
| P3 | -4.54 | 3.74 | -6.60 | 2.77 | -6.36 | 2.60 | -6.34 | 2.50 | -4.33 | 3.13 |
| P4 | -4.79 | 3.45 | -6.24 | 3.24 | -6.23 | 2.69 | -6.39 | 3.05 | -4.19 | 2.80 |
| O1 | -1.14 | 3.57 | -2.48 | 3.55 | -2.20 | 2.84 | -2.22 | 3.04 | -1.01 | 3.36 |
| O2 | -1.13 | 3.49 | -2.54 | 3.28 | -2.33 | 3.23 | -2.36 | 3.19 | -1.02 | 3.33 |
| Theta | | | | | | | | | | |
|  | B1 | | Dog | | Plant | | Replica | | B2 | |
|  | Mean | SD | Mean | SD | Mean | SD | Mean | SD | Mean | SD |
| Fp1 | -1.66 | 3.13 | 0.77 | 4.74 | -0.07 | 2.33 | 1.86 | 3.76 | -1.31 | 3.17 |
| Fp2 | -1.33 | 2.90 | 0.92 | 4.68 | -0.02 | 2.44 | 1.60 | 4.10 | -0.94 | 3.15 |
| F3 | -1.83 | 2.06 | -1.43 | 3.30 | -1.80 | 1.95 | -1.37 | 2.51 | -1.51 | 2.20 |
| F4 | -1.21 | 2.52 | -1.03 | 3.26 | -1.15 | 2.33 | -0.57 | 2.62 | -1.10 | 2.33 |
| C3 | -3.47 | 2.34 | -4.10 | 2.78 | -3.83 | 2.68 | -3.44 | 2.68 | -3.17 | 2.25 |
| C4 | -3.17 | 2.21 | -4.01 | 2.68 | -3.87 | 1.90 | -3.58 | 2.13 | -3.32 | 2.19 |
| P3 | -2.57 | 2.69 | -3.25 | 2.50 | -2.87 | 2.40 | -2.79 | 2.31 | -2.07 | 2.66 |
| P4 | -2.76 | 2.98 | -2.82 | 3.53 | -2.69 | 2.66 | -2.80 | 2.88 | -2.00 | 2.56 |
| O1 | 0.92 | 2.70 | 0.51 | 2.87 | 1.73 | 2.74 | 1.54 | 2.46 | 1.40 | 2.81 |
| O2 | 0.97 | 2.55 | 0.41 | 2.57 | 1.76 | 2.91 | 1.53 | 2.70 | 1.29 | 2.36 |

**Tables S1:** **Descriptive analysis of each frequency band.** Mean and standard deviation (SD) of the power spectral density are reported for each electrode and for all conditions. B1/2=baselines 1/2.
